# Gene editing enables rapid engineering of complex antibiotic assembly lines

**DOI:** 10.1038/s41467-021-27139-1

**Published:** 2021-11-25

**Authors:** Wei Li Thong, Yingxin Zhang, Ying Zhuo, Katherine J. Robins, Joanna K. Fyans, Abigail J. Herbert, Brian J. C. Law, Jason Micklefield

**Affiliations:** Department of Chemistry and Manchester Institute of Biotechnology, The University of Manchester, 131 Princess Street, Manchester, M1 7DN UK

**Keywords:** Synthetic biology, Multienzyme complexes, CRISPR-Cas9 genome editing

## Abstract

Re-engineering biosynthetic assembly lines, including nonribosomal peptide synthetases (NRPS) and related megasynthase enzymes, is a powerful route to new antibiotics and other bioactive natural products that are too complex for chemical synthesis. However, engineering megasynthases is very challenging using current methods. Here, we describe how CRISPR-Cas9 gene editing can be exploited to rapidly engineer one of the most complex megasynthase assembly lines in nature, the 2.0 MDa NRPS enzymes that deliver the lipopeptide antibiotic enduracidin. Gene editing was used to exchange subdomains within the NRPS, altering substrate selectivity, leading to ten new lipopeptide variants in good yields. In contrast, attempts to engineer the same NRPS using a conventional homologous recombination-mediated gene knockout and complementation approach resulted in only traces of new enduracidin variants. In addition to exchanging subdomains within the enduracidin NRPS, subdomains from a range of NRPS enzymes of diverse bacterial origins were also successfully utilized.

## Abstract (web summary)

Engineering biosynthetic assembly lines is a powerful path to new natural products but is challenging with current methods. Here the authors use CRISPR-Cas9 to exchange subdomains within NRPS to alter substrate selectivity.

## Introduction

The provision of new and improved bioactive natural products, particularly anti-infective agents to combat viral pandemics, antimicrobial resistance and treat neglected diseases, is a major global concern^1–4^. Many clinically important natural products, such as vancomycin, daptomycin, penicillin and cephalosporin antibiotics, are derived from highly complex nonribosomal peptide synthetase (NRPS) machineries^5,6^. Derivatizing these highly complex structures to optimize their activity often requires multi-step synthesis, which presents a major barrier in the development of new and urgently required antibiotics^7–9^. Consequently, the development of bioengineering approaches to diversify the core NRPS structures via single-step fermentation, obviating the need for synthetic steps, would be extremely valuable.

NRPS enzymes are comprised of multiple modules, with a minimal set of condensation (C), adenylation (A) and thiolation (T) domains, which are responsible for the activation and incorporation of amino acids into the peptide product. Whilst the modular organization of NRPSs and related megasynthases suggest straightforward approaches for engineering, reprogramming efforts have been met with limited success, as very often these efforts significantly reduce or abolish the activity of the enzymes^10^. Early studies, especially with the clinically important lipopeptide antibiotic daptomycin, have highlighted that for a module swap to be successful the inter-domain and module–module interactions should be maintained, or the resulting chimera will be unproductive^11–13^. The recent identification of conserved domain linker sequences has led to the use of exchange units (XU and XUC) with more versatile and productive fusion points to create chimeric NRPSs^14,15^. Whilst promising, the exchange unit approaches have thus far only been demonstrated with small model NRPSs (up to five modules), assembled and expressed in *Escherichia coli*, producing simple peptides with no bioactivity^14,15^. Many important bioactive nonribosomal peptides are produced in actinobacteria (e.g. *Streptomyces* sp.) by large, complex NRPSs, which are typically not amenable to such engineering and heterologous expression approaches. The high incidence of sequence repeats in NRPSs and the high GC content of many actinobacterial genomes also makes in vivo homologous recombination, using the conventional double-crossover strategy^16^, challenging and can also lead to undesirable genetic rearrangements^17–19^.

An alternative to swapping intact domains is the use of structure-guided active site mutagenesis to alter the selectivity of A domains^20,21^. However, multiple point mutations are often required and only minor changes in final amino acid composition have been reported by this approach^22–25^. A more elegant approach is to swap the highly conserved flavodoxin-like subdomain (FSD) that contains the key active site residues within A domains^26,27^. Exchanging FSD is less likely to perturb the overall structure of the A domain, maintaining the key interactions between other domains in the assembly line. Using this approach, a two-module NRPS (D-Phe-L-Pro) was engineered to produce a D-Val-L-Pro diketopiperazine^27^. This engineered NRPS was also overproduced in *Escherichia coli* and used in vitro to produce the new dipeptide product.

**Fig. 1:**
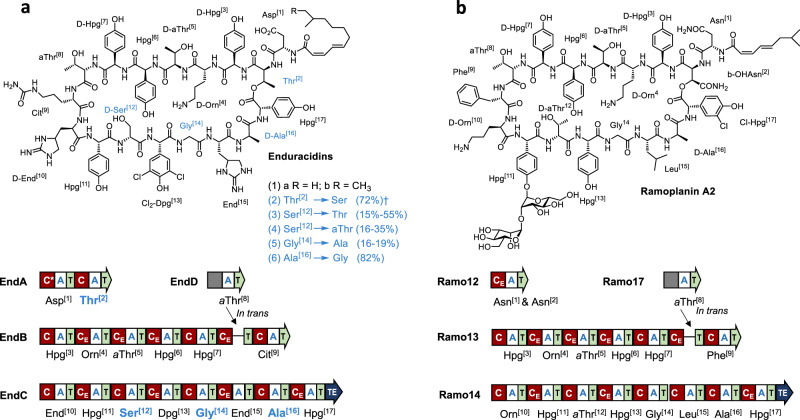
Lipopeptide antibiotic structures and organization of NRPS assembly lines. **a** Structures of wild type and engineered enduracidins; amino acids in blue were targeted for modification, with organization of the enduracidin NRPS shown below. **b** Structure of ramoplanin A2 and organization of the ramoplanin NRPS. Nonproteinogenic amino acid abbreviations: 4-hydroxyphenylglycine (Hpg), ornithine (Orn), *allo*-threonine (*a*Thr), citrulline (Cit), enduracididine (End) and 3,5-dichloro-4-hydroxyphenylglycine (Cl_2_-Dpg), 3-chloro-4-hydroxyphenylglycine (Cl-Hpg). †Titres of engineered products **2**–**6** relative to **1** produced by the wild type *S. fungicidicus*.

To enable biosynthetic engineering of more complex bioactive products, new techniques are required for engineering NRPS assembly lines in the native producer strains. To this end, we envisaged using CRISPR-Cas9 gene editing, adapted for model *Streptomycetes*^28,29^, to introduce a targeted double strand DNA break within an A domain, therefore making subsequent subdomain exchange via homologous replacement more efficient in NRPS genes with highly repetitive sequences. Introducing genetic modifications to native chromosomal loci within intact NRPSs, should enable precursor supply, native regulatory control, tailoring steps and product transport/efflux to be maintained, delivering complex engineered bioactives, which would be challenging using *E. coli* or other heterologous hosts. In this report, we demonstrate how gene editing can be used to engineer the complex 17-module NRPS that produces the lipopeptide antibiotic enduracidin **1** (Fig. 1a)^30^, one of the largest nonribosomal peptides in nature, by efficiently replacing FSDs in vivo at their native loci, without impacting production titres.

## Results

### Adenylation subdomain exchange via CRISPR-Cas9 to generate new enduracidin analogues

**Fig. 2:**
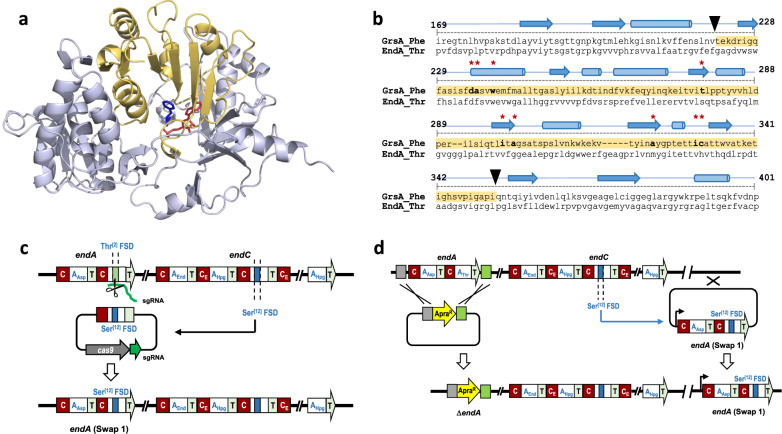
Subdomain swap strategies. **a** The structure of a ligand-bound GrsA Phe-A domain (PDB: 1AMU)^32^. The flavodoxin-like subdomain (FSD) is highlighted in yellow and ligands phenylalanine and adenosine monophosphate (AMP) are shown in blue and red, respectively. Residues HKGISNLKVFFENSLNV which form an alpha helix have been removed for greater visibility of the ligands. **b** Alignment of the *grsA* Phe-A domain with the *endA* Thr^[2]^-A domain, with the secondary structural features indicated and nine of the ten amino acid substrate binding residues (except Lys517) marked with red asterisks^20^. The FSD sequence is highlighted in yellow and the black triangles show cut sites for the subdomain swap. **c** Single-step CRISPR-Cas9 strategy for exchanging subdomains at the native locus. sgRNA-guided Cas9 cleaves within the Thr^[2]^ A domain of *endA*. DNA repair utilizes a plasmid-borne sequence containing *endA* possessing a Thr^[2]^ to Ser^[12]^ subdomain swap, resulting in seamless exchange of the Thr^[2]^ FSD with a Ser^[12]^ FSD at its native locus within the BGC. **d** Schematic for conventional multi step gene knockout and complementation strategy. The wild type *endA* is replaced with an apramycin resistance cassette. The resultant ∆*endA* strain is complemented with an integrative plasmid containing *endA* (Swap 1) where the subdomain sequence of the Thr^[2]^ A domain is replaced with the subdomain from the Ser^[12]^ A domain. Insertion of *endA* (Swap 1) occurs at the *ΦC31* site outside of the enduracidin biosynthetic gene cluster (BGC).

Enduracidin (Fig. 1a) is produced by *Streptomyces fungicidicus* ATCC 21013 and is closely related to ramoplanin (Fig. 1b), from *Actinoplanes* sp. ATCC 33076, which entered phase III clinical trials for the treatment of vancomycin-resistant *Enterococcus*^30,31^. A total synthesis of ramoplanin has been developed, and used to produce variants with improved attributes, but this required over 40 chemical steps^31^, which is not viable for drug development. Biosynthetic engineering approaches for diversification of these lipopeptide antibiotics would be a far more attractive alternative to chemical synthesis. However, genetic manipulation of the huge and repetitive 17-module NRPS systems that deliver these lipopeptide antibiotics would be extremely challenging using the conventional double-crossover homologous recombination method^16^. Enduracidin biosynthesis requires four NRPSs (2.0 MDa) encoded by *endA-D* genes, encompassing 57 kb in total (Fig. 1a). The first of these, EndA, is relatively small and contains just two modules (Fig. 1a). The relatively low complexity of EndA made it a good candidate for initial testing of FSD swaps using CRISPR-Cas9, whilst also allowing comparison under optimal conditions with conventional recombineering methods (Fig. 2). An FSD comprising 139 amino acid residues within the second A domain of EndA that recognizes L-Thr was identified^27,32^, and selected for replacement with the FSD from the L-Ser selective A domain of EndC (Supplementary Fig. 1a). A successful exchange of these two FSDs, which share 88% identity and 89% similarity (Figs. 2a and b, Supplementary Table 2), should result in enduracidin with L-Ser in place of L-Thr at position 2. By making this change at the native chromosomal locus, using the CRISPR-Cas9 system, any disruption to the integrity of the NRPS machinery should be limited and the creation of the mutant strains could be achieved more rapidly than with the laborious multi-step gene knockout/complementation approach.

**Fig. 3:**
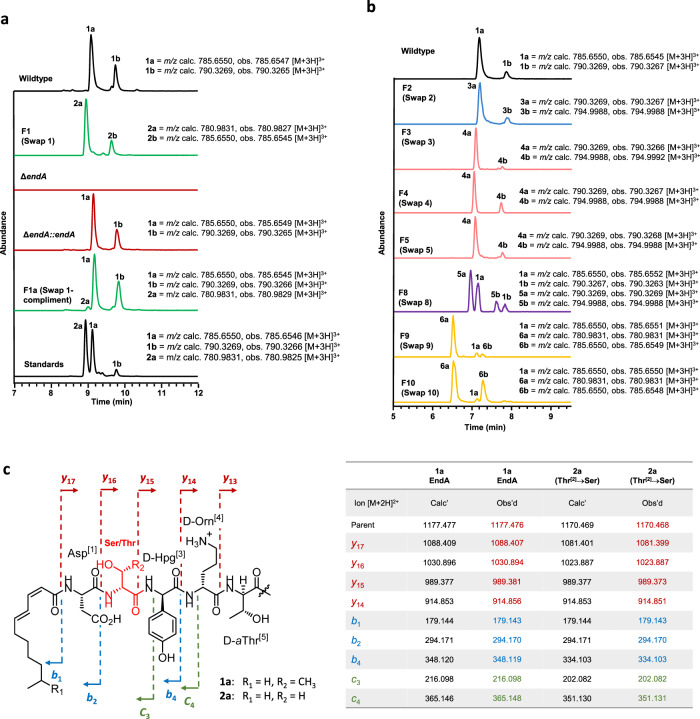
Combined extracted ion chromatographs (EIC) of enduracidin a and b analogues from LC-HRMS analysis of extracts from primary *S. fungicidicus* strains (F1-10) used in this study (normalized to 100%), and tandem mass characterization of 2a. **a** LC-HRMS comparison between CRISPR-Cas9 editing and conventional gene complementation methods for introducing subdomain swaps. The F1a (Swap 1-complement) complementation strain produces mostly **1a** & **1b**, whereas the CRISPR-Cas9 edited strain *S. fungicidicus* F1 (Swap 1) produces Ser-containing enduracidins **2a** & **2b**. **b** LC-HRMS analysis of all productive mutants that generate new enduracidin analogues. **c** LC-HRMS/MS analysis of **1a** and **2a** confirms that Thr has been replaced with Ser in compound **2a**. Fragment ions containing residue 2 are 14 mass units higher in **1a** compared with **2a** (methyl group of Thr). Loss of residue 2 results in fragment ions with identical *m/z* values between **1a** and **2a**.

A pCRISPomyces-2-based plasmid^28^ was constructed, containing a sgRNA that directed a double-stranded DNA cut within the targeted L-Thr FSD and a repair cassette comprised of *endA* with the native FSD exchanged for the alternative L-Ser FSD from *endC* (Supplementary Table 1 and Supplementary Data 1). This repair cassette should repair the resulting DNA break, via homologous recombination, with the copy of *endA* containing the desired FSD change (Swap 1, Fig. 2c, and Supplementary Table 2). The plasmid was introduced into the wild type enduracidin producer, *S. fungicidicus*, resulting in a clean exchange of the original FSD with the new L-Ser selective FSD, at the native locus, as determined by DNA sequencing (Supplementary Fig. 1b). RP-HPLC and LC-HRMS/MS analysis of the resulting mutant strain F1 (Thr to Ser) revealed production of new L-Ser containing variants of enduracidins a and b (**2a** & **2b**) with observed *m/z* 780.9827 (C_106_H_139_C_l2_N_26_O_31_) and 785.6545 (C_107_H_141_C_l2_N_26_O_31_) corresponding to the predicted [M + 3H]^3+^ ions (Fig. 3a) at a high titre of 65 mg/L, with only trace levels of wild type enduracidins (**1a** & **1b**) evident (<3%). The high preference of this strain (>97%) for producing the L-Ser containing enduracidin (**2a** & **2b**), rather than the wild type, confirms that introducing the subdomain change at the native chromosomal locus is very effective. The traces of **1a** & **1b** detected in the mutant strain (F1) could be due to the structural similarity between the amino acids (Thr & Ser). To confirm the structures of the new products, **2a** was purified from strain F1 and compared with **1a** isolated from the wild type *S. fungicidicus* ATCC 21013. Tandem MS analysis of **2a** revealed fragment ions corresponding to a loss of CH_2_ when compared to the equivalent ions of **1a** (Fig. 3c and Supplementary Fig. 2). Fragment ions not predicted to contain the altered amino acid were identical for all four compounds, indicating the observed loss of CH_2_ stems from a Thr to Ser switch at the desired position of enduracidin. The ^1^H NMR spectra of **1a** includes a doublet at 1.39 ppm, corresponding to the methyl moiety of the Thr residue, which was absent in the spectra of **2a** as expected. In addition, a new NOESY correlation signal from the NH moiety of residue 2 at 8.52 ppm to a methylene (CH_2_) signal at 4.06 ppm in **2a**, provides further support that the Thr to Ser exchange (Swap 1) has been successful (Supplementary Figs. 3–6 and Supplementary Table 3).

### A-domain FSD exchange via conventional gene deletion and complementation

Given that *endA* is small, we chose to repeat Swap 1 using a more conventional gene knockout and complementation strategy for comparison with the CRISPR-Cas9 mediated procedure (Fig. 2d). First, we deleted *endA* from the *S. fungicidicus* chromosome by introducing an apramycin resistance cassette *apra*^*R*^ in place of *endA* (Supplementary Fig. 7). The resultant ∆*endA* strain was selected on apramycin-containing media and was unable to produce wild type enduracidins a and b (**1a** & **1b**) (Fig. 3a). A wild type copy of *endA* was introduced back into this strain (forming strain ∆*endA::endA*) under control of the constitutive ermE* promoter, using a vector integrating at the *ΦC31* site, distal to the *end* biosynthetic gene cluster (BGC) locus. This restored production of **1a** & **1b**, albeit at lower levels (14 mg/L) when compared with the wild type strain (108 mg/L). Next, a copy of *endA* engineered with the L-Ser FSD from *endC* (Swap 1), was introduced into the ∆*endA* strain via integration at the *ΦC31* site. Surprisingly, the resulting strain (F1a) still produced wild type enduracidins (**1a** & **1b**) as the major products (9 mg/L), despite possessing a mutant EndA that is identical in sequence to that produced in the CRISPR engineered strain (F1). LC-HRMS analysis revealed additional products with *m/z* 780.9831 and 785.6550 corresponding to the predicted [M + 3H]^3+^ ions of the desired L-Ser containing enduracidin variants (**2a** & **2b**) (Fig. 3a). However, these variants were only observed at very low levels (*ca*. 8% that of **1a** & **1b**, as determined by LC-HRMS). Whole genome sequencing of strain F1a confirmed that the engineered *endA* gene was indeed inserted at a different locus on the chromosome. Given that *endA* would be expressed independently to the native *end* BGC, it is possible that the assembly and interactions of the EndA variant with the other NRPS and gene products of the *end* BGC could be perturbed. This, compounded by the selectivity of the downstream C-domain of EndB and any proofreading machinery, may result in a preference for introduction of L-Thr over L-Ser, especially if peptide assembly is slowed due to poor timing of gene expression and assembly of the NRPS machinery. The fact that the single-step CRISPR-Cas9 strategy was not only more successful in generating a successful amino acid substitution, but did so without a penalty in production titre, indicates that this is a much more efficient way of introducing A domain specificity changes than conventional means. Moreover, the multi-step conventional approaches often leave scars, in this case, an apramycin resistance gene, which can severely limit subsequent genetic manipulation. The CRISPR-Cas9 approach, on the other hand, is scarless, facilitating further strain manipulation, which could potentially enable combinatorial FSD exchanges to be implemented.

### Exploring further FSD changes

**Fig. 4:**
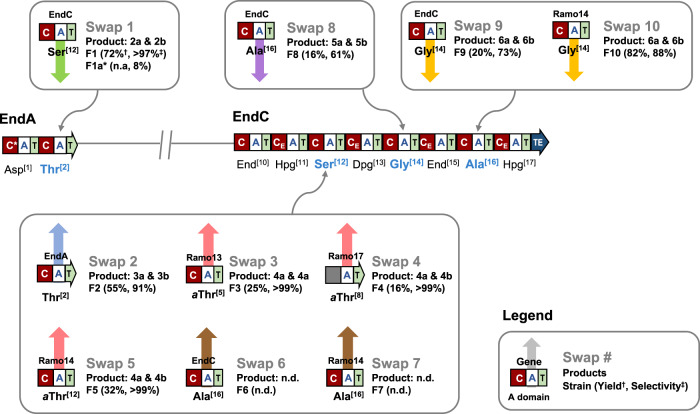
Overview of the primary (Swap 1–10) subdomain swap mutants generated in this study. Most of the subdomain swaps were designed for *endC*, the largest NRPS, containing multiple repeating sequences, which presents a significant challenge for engineering conventional homologous recombination methodologies. *Swap via gene complementation, ^†^ Relative titre of engineered variants (**2–6**) compared titres of **1** produced by wild type, ^‡^ selectivity for new products (**2–6**) over parent enduracidin (**1**) in engineered NRPS. Coloured arrows are used to indicate different amino acid swaps: green (Thr^[2]^ to Ser), blue (Ser^[12]^ to Thr), pink (Ser^[12]^ to *a*Thr), brown (Ser^[12]^ to Ala), purple (Gly^[14]^ to Ala) and yellow (Ala^[16]^ to Gly). Source data for yield and selectivity are provided with this paper.

We next sought to explore the broader applicability of CRISPR-Cas9 subdomain swapping. Genes *endB* and *endC*, encoding six and eight NRPS modules, respectively, are significantly larger than *endA* and contain multiple repeating sequences. Knocking out *endB* or *endC* and complementation with engineered variants, possessing FSD swaps, would present a significant challenge and may not be achievable with standard methodologies. To address this, three modules within EndC, the largest enduracidin NRPS, were targeted for FSD changes by CRISPR-Cas9 (Ser^[12]^, Gly^[14]^ and Ala^[16]^). Bioinformatics analysis of the A domain sequences of both the enduracidin and ramoplanin NRPS^30,33,34^ (Fig. 1) guided the design of nine additional constructs for FSD replacement (Fig. 4 and Supplementary Table 2). First, the complementary exchange to Swap 1, where the L-Ser FSD of EndC was swapped with the L-Thr FSD from EndA, was performed in an analogous fashion. This exchange (Swap 2) resulted in two new enduracidin products **3a** and **3b** with L-Thr in place of the original L-Ser residue at position 12. The change was confirmed by LC-HRMS and LC-HRMS/MS (Fig. 3b and Supplementary Fig. 8). Only trace quantities of wild type enduracidin **1a** and **1b** were evident, with the desired new compounds (**3a** & **3b**) being produced with a selectivity of 91% versus the wild type. To explore if subdomains from a different gene cluster and organism could be introduced, three FSDs from the ramoplanin NRPS that are selective for L-*allo*-Thr were exchanged with the L-Ser FSD of EndC (Swaps 3–5). The selected replacement FSDs were from modules that incorporate *allo*-Thr into positions 5, 8 and 12 of ramoplanin, respectively (Fig. 1)^31,33,34^. All three resulting swaps generated two new products (**4a** & **4b**) consistent with a Ser to *allo*-Thr exchange with high selectivity as little or no **1a** and **1b** could be detected. Although the new products (**4a** & **4b**) have identical masses and MS/MS fragmentation patterns as the Thr containing **3a** and **3b**, LC-HRMS showed shifted retention times as expected for diastereoisomers (Fig. 3b).

Two further exchanges (Swap 6 & 7) were then attempted where the L-Ser FSD of EndC was replaced with Ala-activating FSDs from either EndC or the ramoplanin NRPS Ramo14. However, LC-HRMS analysis showed that these swaps failed to produce the Ala containing variants and production of enduracidin was abolished in both mutants (Supplementary Fig. 9). The FSD swaps in these two strains may have disrupted the activity of the A domain, preventing activation of Ala. Alternatively, strict specificity of the downstream C-domain could have disrupted the processing of the Ala containing variant^14,15,35^. The same Ala FSD from EndC was, however, successfully used to replace the Gly FSD of EndC (Swap 8), resulting in new enduracidin a and b analogues with an Ala in place of Gly (**5a** & **5b**) (Fig. 3b). The opposite replacement of the Ala FSD of EndC with the Gly-activating FSDs from both EndC and Ramo14 (Swap 9 & 10) was also successful and resulted in production of new enduracidin a and b variants consistent with a switch of Ala to Gly (**6a** & **6b**) (Fig. 3b). Production of analogues **5a**, **5b**, **6a** and **6b** was, however, accompanied with 12–39% of the wild type enduracidins **1a** & **1b**. This indicates an incomplete switch in selectivity, which may be due to the close structural similarity between alanine and glycine. To confirm the structures of the new engineered products, the most abundant enduracidin a variants (**3a**, **4a**, **5a** & **6a**) were isolated and subjected to tandem MS analysis. Analysis of fragmentation patterns enabled diagnostic fragmentation ions to be assigned, confirming the positions of the swapped amino acid residues (Supplementary Figs. 8 and 10–15).

**Fig. 5:**
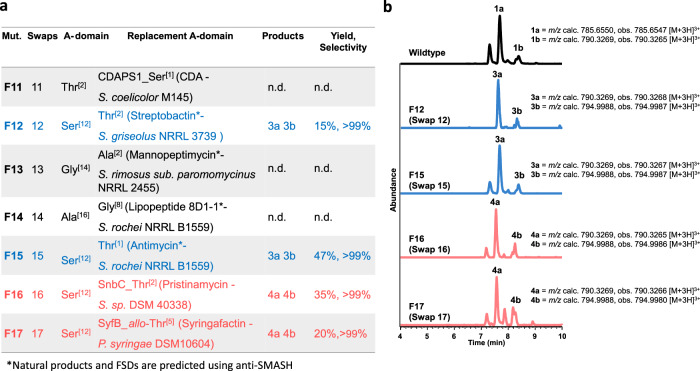
Overview and extracted ion chromatographs (EIC) of enduracidins a and b analogues from LC-HRMS analysis of the additional subdomain swap mutants (F11-17) generated in this study. **a** List of additional swap mutants constructed using FSDs from various NRPSs to demonstrate the broad applicability of this method. **b** Combined EIC of enduracidins a and b analogues from LC-HRMS analysis of extracts from additional *S. fungicidicus* strains (F12 and F15-17) used in this study (normalized to 100%). The product for Swap 16 had a retention time consistent with the incorporation of *allo*-Thr. Source data for yield and selectivity are provided with this paper. *Natural products and FSDs are predicted using anti-SMASH.

To demonstrate the broader applicability of this method, seven additional FSD swap mutants were constructed using diverse NRPS from different bacterial strains (Fig. 5 and Supplementary Table 1). One swap mutant for each of the positions discussed above (Thr^[2]^, Ser^[12]^, Gly^[14]^ and Ala^[16]^) was constructed: Swap 11 (Thr^[2]^ to Ser) used a Ser-selective FSD from the calcium-dependent antibiotic (CDA)^36^ NRPS of *Streptomyces coelicolor* M145; Swap 12 (Ser^[12]^ to Thr) used a predicted Thr activating FSD from a streptobactin-like^37^ BGC in *Streptomyces griseolus* NRRL 3739; Swap 13 (Gly^[14]^ to Ala) employed an FSD predicted to activate Ala from tyrobetaine-like^38^ BGC in *Streptomyces rimosus sub. paromomycinus* NRRL 2455; and Swap 14 (Ala^[16]^ to Gly) was constructed with a predicted Gly-selective FSD from a lipopeptide 8D1-1^39^ BGC found in *Streptomyces rochei* NRRL B1559. Anti-SMASH^40^ was used to identify candidate FSD for swaps 12, 13 and 14 from NRPS that exhibit high similarity to previously well characterized NRPS pathways^37–39^. Among these four mutants, only Swap 12 generated the desired enduracidin variants (**3a** & **3b**) with a selectivity of >99%, with only traces of the wild type enduracidins detected (Fig. 5). Swaps 13 and 14 showed abolished production, while Swap 11 showed traces (<1%) of wild type enduracidin **1a**. The fact that only Swap 12 produces the expected variants is most likely due to the high sequence identity (64%) and similarity (72%) between the FSD that were exchanged. In comparison, the FSD exchanged in Swaps 11, 13, and 14 have lower identity (29–44%) and similarity (40–59%) ranges (Supplementary Table 2).

Based on these results, further FSD exchanges were implemented: Swap 15 (Ser^[12]^ to Thr) using an FSD from an NRPS in *Streptomyces rochei* NRRL B1559, which is predicted by anti-SMASH to produce antimycin^41^; Swap 16 (Ser^[12]^ to Thr) introducing an FSD derived from the known pristinamycin pathway in *Streptomyces sp*. DSM 40338^42^; and finally Swap 17 (Ser^[12]^ to *allo*-Thr) using an FSD from a BGC of *Pseudomonas syringae* DSM 10604 that is known to produce syringafactin^43^. All three mutants generated new enduracidin analogues, which was expected based on the high sequence similarity between the FSDs that are exchanged (Supplementary Table 2). Swap 15 produced variants **3a** & **3b** and Swaps 16 and 17 gave the compounds **4a** & **4b** at >99% selectivity (Fig. 5). Of the 17 FSD exchanges that were performed, 12 were successful in producing enduracidin variants. This provides a guideline for future engineering, with successful swaps occurring between FSD that have an identity of >55% and a similarity of >65% (Supplementary Table 2).

### Effect of FSD changes on production titre

**Fig. 6:**
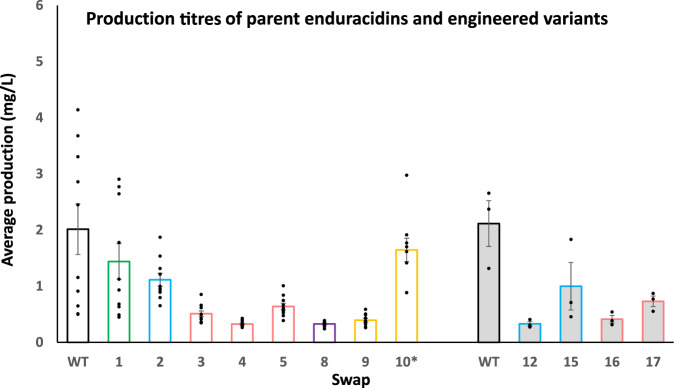
Titres of enduracidin analogues produced by FSD swap mutant. Production levels (mg/L) for engineered enduracidin from FSD swaps compared with the wild type enduracidin (**1a** & **1b**). Non-filled bars represent a batch of ten replicates (*n* = 10) for Swaps 1–5 and Swaps 8–10. Shaded bars represent a different batch of cultures carried out in triplicate (*n* = 3) for Swaps 12, 15, 16 and 17. Data are presented as mean values + /− standard error. Each data point is overlaid on the graph as a dot plot. The wild type strain is included in both batches as a positive control. The production for Swap 10 was calculated from an average of eight samples, omitting two statistical outliers from flasks 1 and 5. The ratio of enduracidin a & b variants produced by wild type and engineered strains is variable and so the combined yields (a & b) are reported in each case to enable a clearer comparison of the productivity of each strain. Each bar is colour-coded for the analogues produced: green (**2a** & **2b**), blue (**3a** & **3b**), pink (**4a** & **4b**), purple (**5a** & **5b**) and yellow (**6a** & **6b**). Source data are provided with this paper.

In order to quantify the effects of the subdomain changes on overall enduracidin titre, we cultivated all the subdomain swap mutants and the wild type *S. fungicidicus* under identical conditions. *Streptomyces* strains are known to be highly susceptible to variations in culture conditions; therefore ten replicates per strain were prepared and were cultured simultaneously to minimize disparities in culture time or temperature fluctuations. To accommodate parallel cultivation of a large number of strains and replicates, the fermentation conditions were optimized for higher throughput, which involved changing media components and reducing the volume of cultures. When grown under these new conditions, the wild type *S. fungicidicus* produced significantly lower enduracidin titres of 2.02 ± 0.45 mg/L (**1a** & **1b**) when compared to the previous conditions that were optimized for production of **1a** and **1b** (108 mg/L) (Fig. 6, Supplementary Fig. 16 and Supplementary Table 4). However, these new conditions did allow a reliable comparison between strains to be made. Notably, several mutant strains produced enduracidin variants in yields approaching that observed by the wild type; strain F1 (Thr^[2]^ to Ser, Swap 1), F2 (Ser^[12]^ to Thr, Swap 2), and F10 (Ala^[16]^ to Gly, Swap 10) produced variants (**2a** & **2b**), (**3a** & **3b**) and (**6a** & **6b**) in titres of 72%, 55% and 82% relative to the wild type. The three strains (F3–F5) containing Ser^[12]^ to *allo*-Thr exchanges (Swaps 3–5) produced variant **4a** and **4b** in lower relative yields (16–32%) compared with the wild type. Strains F8 (Gly^[14]^ to Ala, Swap 8) and F9 (Ala^[16]^ to Gly, Swap 9) also gave reduced yields of 16–20% (Fig. 4, Fig. 6, and Supplementary Table 4). The strains F12 and F15–F17 which were generated from FSD derived from NRPS produced by more diverse *Streptomyces* and *Pseudomonas* species, gave similar yields of engineered enduracidins (**3a** & **3b** or **4a & 4b**), in the range 16% to 47%, relative to the wild type strain (Fig. 6 and Supplementary Table 4).

### Whole genome sequencing to evaluate off-target effects of CRISPR-Cas9 editing

In order to establish if the CRISPR-Cas9 gene editing caused any off-target effects that may have impacted on the mutant strains ability to produce enduracidin, six strains were subjected to PacBio whole genome sequencing. The wild type strain was also sequenced, and the mutant strains were aligned with this to identify any off-target mutations. Mutant strains with a range of enduracidin production levels were sequenced, from relatively high levels of production (F1, F2, and F10) to no production (F7). All of the mutant strains had the correct swapped subdomains, as previously confirmed by DNA sequencing. In addition, all the strains sequenced did not possess any mutations anywhere in the enduracidin gene cluster, which indicates that CRISPR-Cas9 gene editing of NRPS does not lead to any of the rearrangements that have been seen with genetic manipulations of other genes encoding related megasynthases^17–19^. Most mutant strains did have a number of single-nucleotide variants (SNVs) or small insertions or deletions (indels) (Supplementary Table 5). The three strains that had the highest titres of enduracidin production (F1, F2, and F10) did exhibit the lowest number of mutations across their genomes, with F2 having no mutations at all other than the FSD swap. The strain that produced no enduracidin (F7) also had a relatively low number of mutations (2 indels and 1 SNV), ruling out off-target effects of the CRISPR-Cas9 editing in explaining the lack of production in this strain. The other two strains (F8 and F9) sequenced had relatively low enduracidin production levels and a higher number of mutations within the genome (14 mutations for F8, and seven for F9), although none of these can explain the lower levels of enduracidin produced and so it is most likely that the swapped subdomain has affected the efficiency of these NRPSs.

## Discussion

In this study, we show how CRISPR-Cas9 gene editing can be used to engineer highly complex NRPS assembly lines, which to the best of our knowledge, has not been described previously. Several of the engineered NRPSs were highly selective for non-cognate amino acid substrates, producing new lipopeptides in titres approaching the wild type strain. We observed that FSDs with identical selectivity, but from different origins, can have an effect on the production titre of the system as Swap 9 resulted in only 25% of the production of Swap 10 despite both FSDs selecting an identical amino acid (Fig. 4). Whilst the reasons for such variations are not clear, subtle differences in sequence and structure between subdomains may make some FSDs more compatible than others. Nevertheless, the high speed, efficiency and fidelity of CRISPR-Cas9 mediated replacements, compared to conventional methods, makes the screening of multiple FSD exchanges much more feasible, significantly increasing the scope to create optimized engineered assembly lines. Whole genome sequencing of six of the strains generated here indicate very accurate FSD swaps, with no other mutations introduced into the BGC or other pathways that are essential for antibiotic production. Moreover, the improvements seen with CRISPR-Cas9 compared to an identical switch using the conventional gene knockout/complementation approach also shows the benefits of this new approach.

Previously, CRISPR-Cas9 gene editing has been used to delete genes from BGC, to knock-in strong promoters for the activation of silent BGC and to enable in vitro engineering of PKS encoding genes for heterologous expression^44–49^. Here we demonstrate how CRISPR-Cas9 gene editing can be used to efficiently swap subdomains within large NRPS, in the native host, leading to engineered variants of complex lipopeptide antibiotics that would be extremely demanding to prepare synthetically. Our approach overcomes some of the limitations inherent in other NRPS engineering methods that rely on utilizing a model heterologous host, such as *E. coli*, where functional expression of large NRPS products can be problematic^47^. Our methodology is rapid, does not leave scars, and has the potential to be implemented in a combinatorial fashion. We therefore envisage that this method could be used to alter the sequences of a wide range of complex nonribosomal peptides in native hosts, “fine-tuning” their bioactivity and other properties. Although the scope of adenylation domain engineering may be limited by the specificity of downstream C-domains, the combination of our gene editing method with other NRPS engineering approaches, including new methods of modulating C-domain selectivity^15^, may allow for more significant changes in peptide structure to be realized. Additionally, the direct editing of gene clusters on the native chromosome means larger NRPS genes, such as *endC*, can now be targeted for engineering, which may not have been possible with conventional methods. Finally, we envisage that the gene editing approach described in this study could also be employed to re-programme other native pathways, including polyketide synthase (PKS) and hybrid NRPS-PKS assembly lines, which are also a prolific source of antibiotics and other therapeutic agents.

## Methods

### General fermentation of *S. fungicidicus* ATCC 21013 wild type and mutant strains, extraction and analysis of enduracidins

*Streptomyces fungicidicus* spores were used to inoculate tryptone soy broth (TSB) media and the seed cultures cultivated at 30 °C for 2–3 days until a high cell density was reached. For fermentation, the seed culture (1.5 mL) was inoculated into a 250 mL Erlenmeyer flask containing 30 mL of enduracidin production media (3% (v/v) corn steep liquor (Sigma-Aldrich), 2% soybean flour (Holland & Barrett), 0.5% NaCl, 1% CaCO_3_, 2% glucose, 5% soluble starch (Sigma-Aldrich), adjusted to pH 7.0 prior to autoclaving). The fermentation cultures were incubated with 180 rpm agitation at 30 °C for 12–14 days.

For enduracidin crude extraction and analysis, the mycelia were pelleted by centrifugation and extracted with three volumes of acidic methanol (~pH 2.0). The methanol extracts were centrifuged and the supernatant analysed by RP-HPLC on a Shimadzu UFLC XR HPLC system with a Phenomenex Aeris XB-C18 peptide column 150 × 4.6 mm, 3.6 µm particle size, UV detection wavelength 267 nm, flow rate 1.5 ml min^−1^ with the following gradient: 0–1 min 10% B, 1–1.5 min 10–30% B, 1.5–11.5 min 30–50% B, 11.6–13 min 95% B, 13.1–15 min 10% B, where mobile phase A is H_2_O + 0.1% formic acid and mobile phase B is methanol + 0.1% formic acid. HPLC data analysis was performed using Shimadzu LabSolution Lite v5. LC-HRMS and MS/MS analyses of enduracidins were carried out on a Thermo Dionex Ultimate 3000 UHPLC coupled to a Thermo QExactive mass spectrometer, using a Thermo accucore C18 column, 100 × 2.1 mm, 2.6 µm particle size, flow rate 0.3 mL min^−1^, with the following gradient: 0–1 min 10% B, 1–1.5 min 10–25% B, 1.5–9 min 25–60% B, 9–9.2 min 60–95% B, 9.2–10.5 min 95% B, 10.5–11 min 95–10% B, 11–12.5 min 10% B, where mobile phase A is H_2_O + 0.1% formic acid and mobile phase B is methanol + 0.1% formic acid. For LC-MS/MS analysis an inclusion list was used to target compounds for fragmentation, with CID 25, 30, 35 eV. LC-MS data analysis was performed using Thermo Xcalibur v2.2, and ChemDraw Professional v16 was used for exact mass calculations.

### Isolation and NMR analysis of enduracidin a (1a) and analogue (2a)

Crude methanol extracts containing enduracidin were prepared as described previously. The methanol was removed under reduced pressure leaving a crude acidic aqueous extract that was extracted with two equal volumes of ethyl acetate. The aqueous layer was then adjusted to pH 7–8, and the enduracidin was extracted into butanol (two volumes). The butanol layers were pooled and the solvent removed under reduced pressure, leaving a yellow–brown solid. This was dissolved in 50% methanol and purified using semi-preparative RP-HPLC as follows: Shimadzu Prominence HPLC with a Phenomenex Gemini C18 column 250 × 10 mm, 5 µm particle size, UV detection wavelength 267 nm, flow rate 5 ml min^−1^, with the following gradient: 0–2 min 10% B, 2–18 min 10–60% B, 18–19 min 60–95% B, 19–21 min 95% B, 21–22 min 95–10% B, 22–25 min 10% B, where mobile phase A is H_2_O + 0.1% formic acid and mobile phase B is methanol + 0.1% formic acid. Fractions containing the desired enduracidins were collected, pooled and the methanol removed under reduced pressure. The residual water fraction was frozen, and the water was removed by lyophilization to leave pure enduracidin. For NMR analysis, the samples were dissolved in H_2_O:D_2_O:TFA 9:1:0.05 (v/v/v) and was performed on a B500 Bruker Avance 500 MHz NMR spectrometer. Previously reported NMR data of enduracidin was used as a reference in our NMR analysis of enduracidin **1a** and analogue **2a**^50^. NMR data analysis was performed using MestReNova v11.

### Quantification of enduracidin a and b by *S. fungicidicus* wild type and new variants by mutant strains

To quantify the production of our new enduracidin versus wild type enduracidin, we cultivated all mutants and the wild type in replicate flasks. The mycelia were pelleted by centrifugation and extracted twice, first with three volumes of methanol followed by three volumes of acidic methanol (~pH 2.0). The methanol was then removed under reduced pressure and the remaining acidic crude aqueous extract was adjusted to pH 7–8, frozen and lyophilized to yield a dry crude extract. The extracts were then reconstituted with methanol and analysed by LC-HRMS under the same conditions as stated previously. Titres of target compounds were calculated based on the peak area of the extracted ion chromatogram of each enduracidin a and b analogue in relation to the standard curve (Supplementary Fig. 16) of a commercial standard containing enduracidin **1a** and enduracidin **1b** (Toku-E).

### Deletion of *end* ORFs in *S. fungicidicus* ATCC 21013

Deletion of the *endA* gene was carried out using homologous recombination with a plasmid constructed using REDirect PCR targeting^47^. A 1.5 kb homologous region upstream of *endA* were PCR amplified from *S. fungicidicus* ATCC 21013 genomic DNA using primers JKF_JM_1F(5ʹ-GCGCCTCGAGTCGCTATGTGTCGCGTCGCT-3ʹ) and R(5ʹ-GCGCGAATTCTCCAGGTCCTTTCGACGCAT-3ʹ), and inserted into a pBluescript KS + based vector, pDWU5 (a gift from David Widdick, John Innes Centre, Supplementary Table 1), using XhoI and EcoRI restriction sites. Next, a 1.5 kb homologous region downstream of *endA* was amplified using primers JKF_JM_3F(5ʹ-GCGCGAATTCCGCTGCGCCCGATGCGCTGA-3ʹ) and R(5ʹ-GCGCTCTAGAAACCCCTCACCAGCCGCGA-3ʹ) and inserted using restriction sites EcoRI and XbaI. The resultant plasmid pDWU5-∆*endA* containing upstream and downstream *endA* flanking arms was transformed into *E. coli* BW25113/pIJ790.

An apramycin resistance cassette *apra*^*R*^ was amplified from pIJ773 using primers endA_RED_F(5ʹ-CTGACAGCCGACTGAGGGCCTTCGAGGGGGAGGACGATGATTCCGGGGATCCGTCGACC−3ʹ) and R(5ʹ-GCGAACGACGCGGGAATCATGTGGAACCCCTTTCCTTCATGTAGGCTGGAGCTGCTTC−3ʹ) to produce an oriT-FRT-*apra*^*R*^-FRT fragment that was subsequently inserted into plasmid pDWU5-∆*endA* using PCR targeting, generating plasmid pDWU5-∆*endA-apra*^*R*^. Introduction of the plasmid into *S. fungicidicus* ATCC 21013 was carried out by conjugal transfer from *E. coli* ET12567/pUZ8002. Ex-conjugant single crossover colonies (apramycin resistant and kanamycin resistant phenotypes) were selected on SFM medium plate containing apramycin and kanamycin. Double-crossover colonies containing the *endA* deletion were selected for apramycin resistant but kanamycin sensitive phenotypes. Confirmation of *endA* deletion was carried out by PCR (Supplementary Fig. 7).

### Complementation of the ∆*endA* strain with wild type endA

The wild type *endA* gene was amplified from *S. fungicidicus* ATCC 21013 genomic DNA in two parts. Prior to cloning the *endA* gene, the expression promoter (ermE*p) sequence was excised from pIJ86 and inserted in pDWU5 using XbaI/BamHI to generate plasmid pDWU5-endA2-1. Then, the first half of *endA* was amplified from genomic DNA using primers JKF_JM_5 F(5^'^-CGGCGGCCAGCTCGAGTTCG-3ʹ) and R(5^'^-GCGCGGTACCGAATTCTGGGGCTCGCCG-TCGGCTTC-3ʹ) and inserted into pDWU5-endA2-1 using XhoI and BamHI restriction sites to generate plasmid pDWU5-endA2-2. The second half of *endA* was amplified using primers JKF_JM_7 F(5ʹ-GCGCGGATCCCTTCGAGGGGGAGGACGATG-3ʹ) and R(5ʹ-CCCGCGAACTCGAGCTGG-3ʹ) and subcloned into pDWU5 using BamHI/XhoI to generate plasmid pDWU5-endA1-2. The ermE*p-endA sequence from plasmid pDWU5-endA2-2 was excised using XbaI and XhoI and inserted into pDWU5-endA1-2 to generate pDWU5-endA, containing the full length *endA* sequence. Due to the *ΔendA* strain containing an *apra*^*R*^ cassette in place of *endA*, the *apra*^*R*^ of plasmid pDWU5-endA was replaced with a hygromycin resistance cassette *hyg*^*R*^. The *hyg*^*R*^ sequence was amplified from pIJ10700 using primers HygR F(5ʹ-GATCGACTGATGTCATCAGCGGTGGAGTGCAATGTC-3ʹ) and R(5ʹ-GCCCCTCCAACGTCATCTCGTTCTCCGCTCATGAGCTCATCAGGCGCCGGGGGCGGTGTCCG-3ʹ). The *hyg*^*R*^ fragment was inserted into pDWU5-endA using REDirect PCR targeting.

### Complementation of the ∆*endA* strain with *endA*(Ser^[12]^FSD)

A section of *endA* immediately upstream of the Thr^[2]^ FSD, was amplified using primers JKF_JM123 F (5ʹ-GCGCAAGCTTTCTAGAAATCCCGCCTTCGACGACCTCC-3ʹ) and R(5ʹ-GCGCGATATCGAACTCGAACACCCCCCGGG-3ʹ) and inserted into pDWU5 using HindIII/EcoRV restriction sites to generate plasmid pDWU5-endA-1. Subsequently, the region of *endA* downstream of the Thr^[2]^ FSD was amplified using primers JKF_JM125F(5ʹ-GCGCGATATCCCGGGGTTGTCGGTGTTCCTG-3ʹ) and R(5ʹ-GCGCGAATTCTCTAGATGGGGCTCGCCGTCGGCTTC-3ʹ) and inserted into the plasmid pDWU5-endA-1 using EcoRV/EcoRI to generate plasmid pDWU5-endA-2. The middle part of *endA* was amplified from genome by using primer JKF_JM_149F(5'-CTGGACCGCCGGCGGCCAGCTCGAGTTCGCGGGCCGGGCCGAC-3') and R(5'-TCGCATCAGGCGTCCTCGCCGCCTGCACC-3'). The final section of *endA* without Thr^[2]^ FSD coding sequence was amplified from pDWU5-endA-2 by using primer JKF_JM_151F(5'-GGCGAGGACGCCTGATGCGACGGCGGTGAC-3') and R(5'-AGCTGGGTACCGGGCCCCCCGAATTCTGGGGCTCGCCGTCGGCT-3'). The two PCR fragments, the middle part of *endA* and final part of *endA*, were inserted into plasmid pDWU5-endA1-2 by HiFi assembly resulting in the plasmid pDWU5-ermE*p-endA-no-Thr^[2]^FSD. The apramycin resistance gene in pSET152 will be replaced with hygromycin resistance gene from pIJ10700 by PCR targeting (REDirect) using primers apra2hygro_F(5'-GATCGACTGATGTCATCAGCGGTGGAGTGCAATGTC-3') and R (5'-GCCCCTCCAACGTCATCTCGTTCTCCGCTCATGGCTCATCAGGCGCCGGGGGCGGTGTCCG-3') to create plasmid pSET152-hygR. The ermE*p::endA-no-Thr^[2]^ FSD fragment was amplified using primer JKF_JM_213F(5'-CTCTAGAGGATCCGCGGCCGCGCGCGAATTCGAGCTCGGTACCAGC-3') and R(5'-ACAGCTATGACATGATTACGAATTCGAATTCTGGGGCTCGCCG-3') and inserted into the EcoRV site of pSET152-hygR by HiFi assembly to give pSET152-hygR::ermE*p::endA-no-Thr^[2]^FSD. The Ser^[12]^FSD_endC was amplified with primers JKF_JM_127F(5'-CCCGGGGGGTGTTCGAGTTCGGAGCAGACGACGTGTGGAGCGGCTTCC-3') and R(5'-CAGGAACACCGACAACCCCGGCAGACCCCGCCCGATCACGCTGCCGTC-3') from genomic DNA of *S. fungicidicus* and inserted into the EcoRV site of the plasmid pSET152-hygR::ermE*p::endAnoThr^[2]^FSD to generate plasmid pSET152-hygR::ermE*p::endA-no-Thr^[2]^SD-Ser^[12]^SD.

### CRISPR-Cas9 editing: plasmid construction, conjugation and validation of subdomain replacement mutants

Primers used in cloning CRISPR-Cas9 constructs are listed in Supplementary Data 1. Sequence alignments were performed using Clustal OMEGA web form and Vector NTI Suite v6. Synthetic protospacer sequences designed to cut within the subdomain sequence were synthesized by Genewiz, and inserted into pCRISPomyces-2 vector as reported in Cobb et. al. 2015^28^. Upstream and downstream ~2–2.4 kb homology arms flanking each target A subdomains were amplified from *S. fungicidicus* genomic DNA using respective primers listed. By using HiFi assembly (NEB), these flanking arms were then inserted into the protospacer-containing pCRISPomyces-2 vector, which has been linearized at the XbaI site. Subsequently, subdomains for each corresponding exchange were amplified from genomic DNA using primers listed and inserted into the EcoRV site located between the two homology arms, generating each construct. All constructs were verified by restriction enzyme digestion and nucleotide sequencing. Introduction of each sequence verified-construct into *S. fungicidicus* was performed via intergeneric conjugation. These plasmids were first transformed into *E. coli* ET12567 /pUZ8002 and the resultant strains were used in subsequent conjugations following standard procedures^16^. All ex-conjugants formed (typically after 14 days of cultivation) were picked and streaked onto SFM plates supplemented with nalidixic acid (25 µg/mL) and apramycin (50 µg/mL). Colonies that grow after 7 days of incubation at 30 °C were then screened for successful subdomain exchange by colony PCR, and correct mutants were cultivated at high temperature (39 °C) to promote loss of the plasmid. Colonies that grow after the high-temperature incubation (~7 days), were screened again by replicate plating onto SFM with and without apramycin. Finally, successful subdomain swapping on colonies that have lost apramycin resistance were confirmed by colony PCR and nucleotide sequencing.

### Whole genome sequencing of selected mutants

Wild type *S. fungicidicus* as well as strains F1, F2, F7, F8, F9 and F10, were subject to whole genome sequencing. gDNA was isolated using NEB Monarch® Genomic DNA Purification Kit. The gDNA was then sheared using a gTube (Covaris, roughly 10 kb fragments) and prepared for sequencing following the SMRTbell Express Template Preparation Kit 2.0 protocol. Sequencing data were obtained using the Sequel system (Pacific Biosciences) with a 10 h acquisition. Assembly of the genome was performed using the HGAP4 algorithm in SMRT Link v8.0. Assembled genomes were aligned using mauve multiple genome alignment with Geneious Prime 2021.1.

### Reporting summary

Further information on research design is available in the Nature Research Reporting Summary linked to this article.

## Supplementary information

Supplementary Information

Description of Additional Supplementary Files

Supplementary Data 1

Reporting Summary

## Data Availability

All whole genome sequencing data generated in this study have been deposited in the NCBI genome database under BioProject PRJNA776125, samples SAMN22746534-SAMN22746540. The remaining data generated or analysed during this study are included in the Article, Supplementary Information, Supplementary Data file and Source Data file. Nucleotide sequences were obtained from GenBank and their accession numbers are as follows: enduracidin (DQ403252); ramoplanin (DD382878); CDA (CAB38518); pristinamycin (CAA67248); all other subdomain sequences are included in the Supplementary Methods. The original materials such as plasmids and strains that support the findings of this study are available from the corresponding author upon reasonable request. Source data are provided with this paper.

## Source data

Source Data
