# Supplementary material for: Gene editing enables rapid engineering of complex antibiotic assembly lines
Source: Nat Commun. 2021 Nov 25;12:6872. doi: 10.1038/s41467-021-27139-1 (PMC8616955; doi:10.1038/s41467-021-27139-1)
Supplement: Supplementary file 1 — Supplementary Information [file 41467_2021_27139_MOESM1_ESM.pdf]

Supplementary Information

Gene editing enables rapid engineering of complex antibiotic assembly lines.

Authors: Wei Li Thong†, Yingxin Zhang†, Ying Zhuo†, Katherine J. Robins, Joanna K. Fyans, Abigail J. Herbert, Brian J.C. Law, and Jason Micklefield*.

Department of Chemistry and Manchester Institute of Biotechnology, The University of Manchester, 131 Princess Street, Manchester M1 7DN, UK.

†These authors contributed equally.

*Corresponding author Email: jason.micklefield@manchester.ac.uk

Table of Contents

**Supplementary Fig. 1**	Sequence alignments for the swap 1 constructs
**Supplementary Fig. 2**	Characterisation of **2a** produced in this study
**Supplementary Fig. 3**	1H 1D spectra of **1a** (a) and **2a** (b) in H_2O-D_2O-TFA (9:1:0.05, v/v/v), acquired at 500 MHz
**Supplementary Fig. 4**	2D NOESY spectra of **1a** (a) and **2a** (b) in H_2O-D_2O-TFA (9:1:0.05, v/v/v), acquired at 500 MHz
**Supplementary Fig. 5**	Enlarged section of the 1H NMR spectra of **1a** and **2a**
**Supplementary Fig. 6**	Enlarged section of the NOESY spectra of **1a** and **2a**, focusing on the NH moiety of residue 2
**Supplementary Fig. 7**	Homologous recombination mediated *endA* gene knock-out and gene complementation
**Supplementary Fig. 8**	Extracted ion chromatographs from LC-HRMS analysis of extracts from *S. fungicidicus* strains used in this study
**Supplementary Fig. 9**	LC-HRMS/MS characterisation of **3a** produced by mutant F2 in this study
**Supplementary Fig. 10**	LC-HRMS/MS characterisation of **4a** produced by mutant F3 in this study.
**Supplementary Fig. 11**	LC-HRMS/MS characterisation of **4a** produced by mutant F4 in this study.
**Supplementary Fig. 12**	LC-HRMS/MS characterisation of **4a** produced by mutant F5 in this study.
**Supplementary Fig. 13**	LC-HRMS/MS characterisation of **5a** produced by mutant F8 in this study.
**Supplementary Fig. 14**	LC-HRMS/MS characterisation of **6a** produced by mutant F9 in this study.
**Supplementary Fig. 15**	LC-HRMS/MS characterisation of **6a** produced by mutant F10 in this study.
**Supplementary Fig. 16**	Calibration curve of commercial enduracidin **a** standard, plotted based on the peak area of the extracted ion chromatogram of m/z 785.6550 $[M+H]^{+.}$
**Supplementary Table 1**	Bacterial strains and plasmids used in this study
**Supplementary Table 2**	Bioinformatics information for each swap mutant
**Supplementary Table 3**	Key proton chemical shifts (ppm) that identify each residues in enduracidin **a** and its new analogue.
**Supplementary Table 4**	Yield of enduracidin (**1a** & **1b**) and engineered variants in each cultivation flasks as estimated based on the standard curve of commercial enduracidin standard.
**Supplementary Table 5**	Mutations identified via whole genome sequencing
**Supplementary Methods**	
**References**	

Supplementary Figures

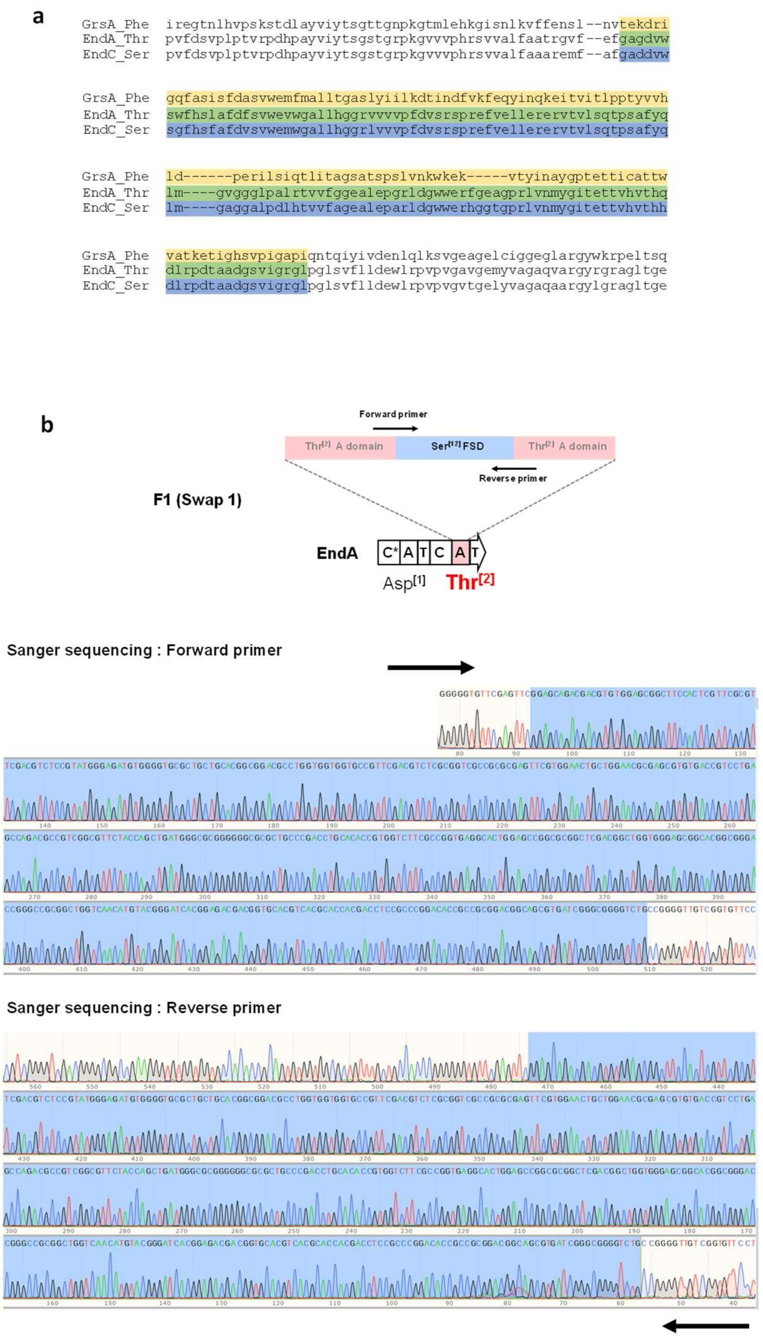

Supplementary Fig. 1. Sequence alignments of the subdomain for the swap 1 constructs. a, Alignment of the GrsA Phe-A domain with the *endA* Thr[2] and *endC* Ser[12] A domains, with the subdomain regions highlighted. **b**, An example sequencing result for the subdomain of mutant F1 showing the swap 1 has occurred.

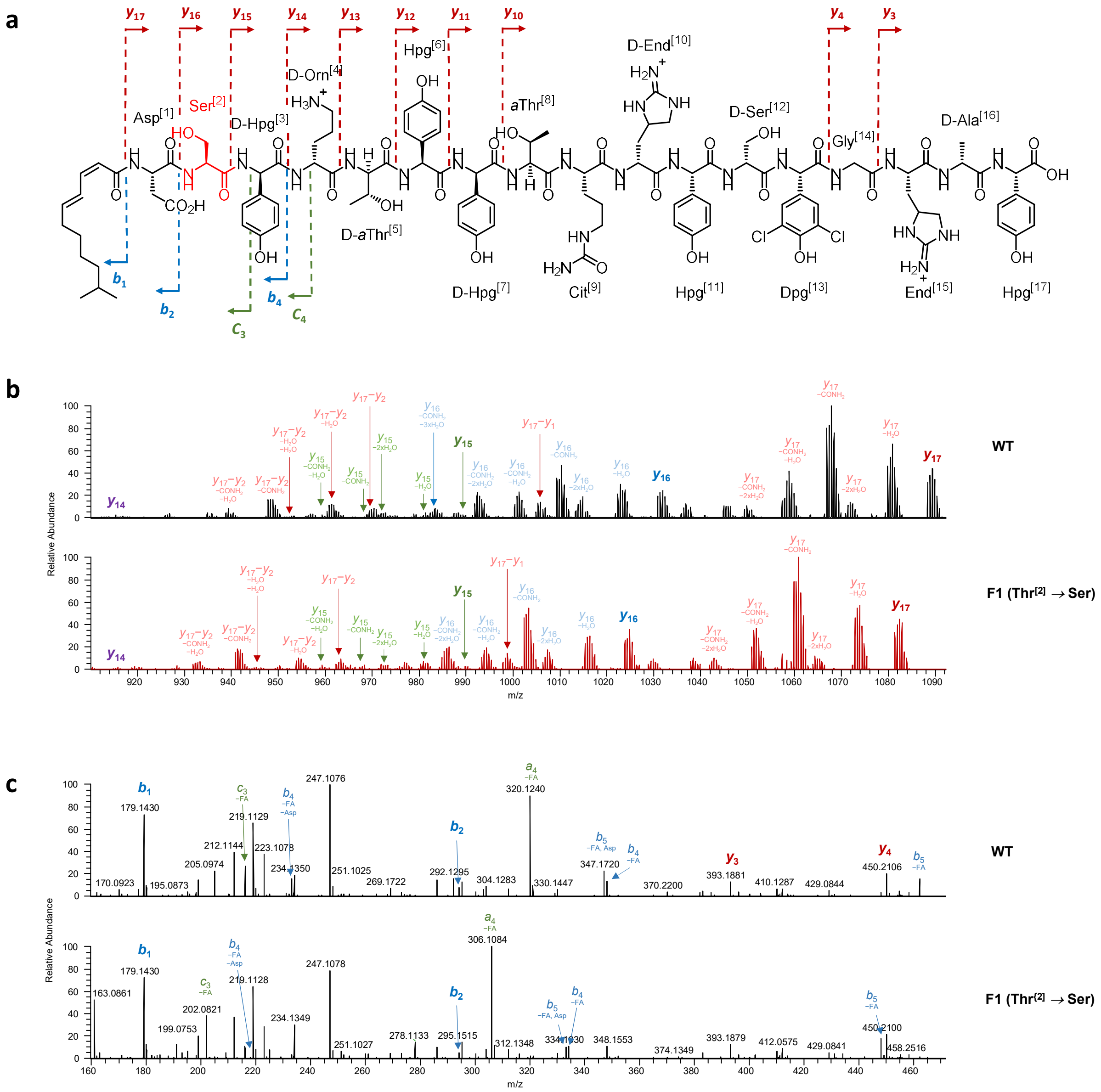

Supplementary Fig. 2. Characterization of 2a produced in this study. a, MS/MS fragmentation observed in **2a**. **b**, MS/MS fragmentation of *y* ions. **c**, MS/MS fragmentation of *b* and *c* ions.

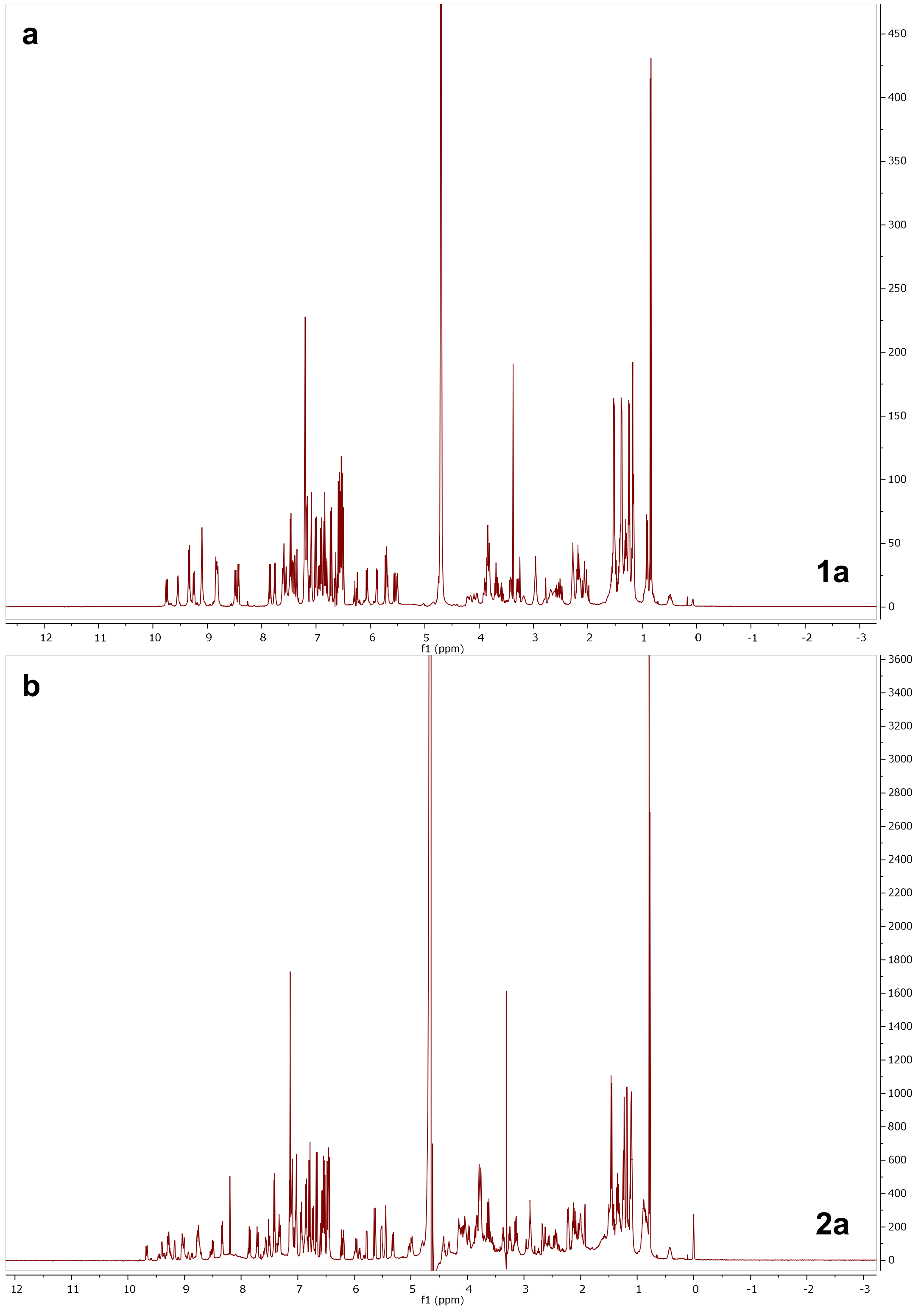

Supplementary Fig. 3. ^{1}H 1D spectra of 1a (a) and 2a (b) in H_2O-D_2O-TFA (9:1:0.05, v/v/v), acquired at 500 MHz.

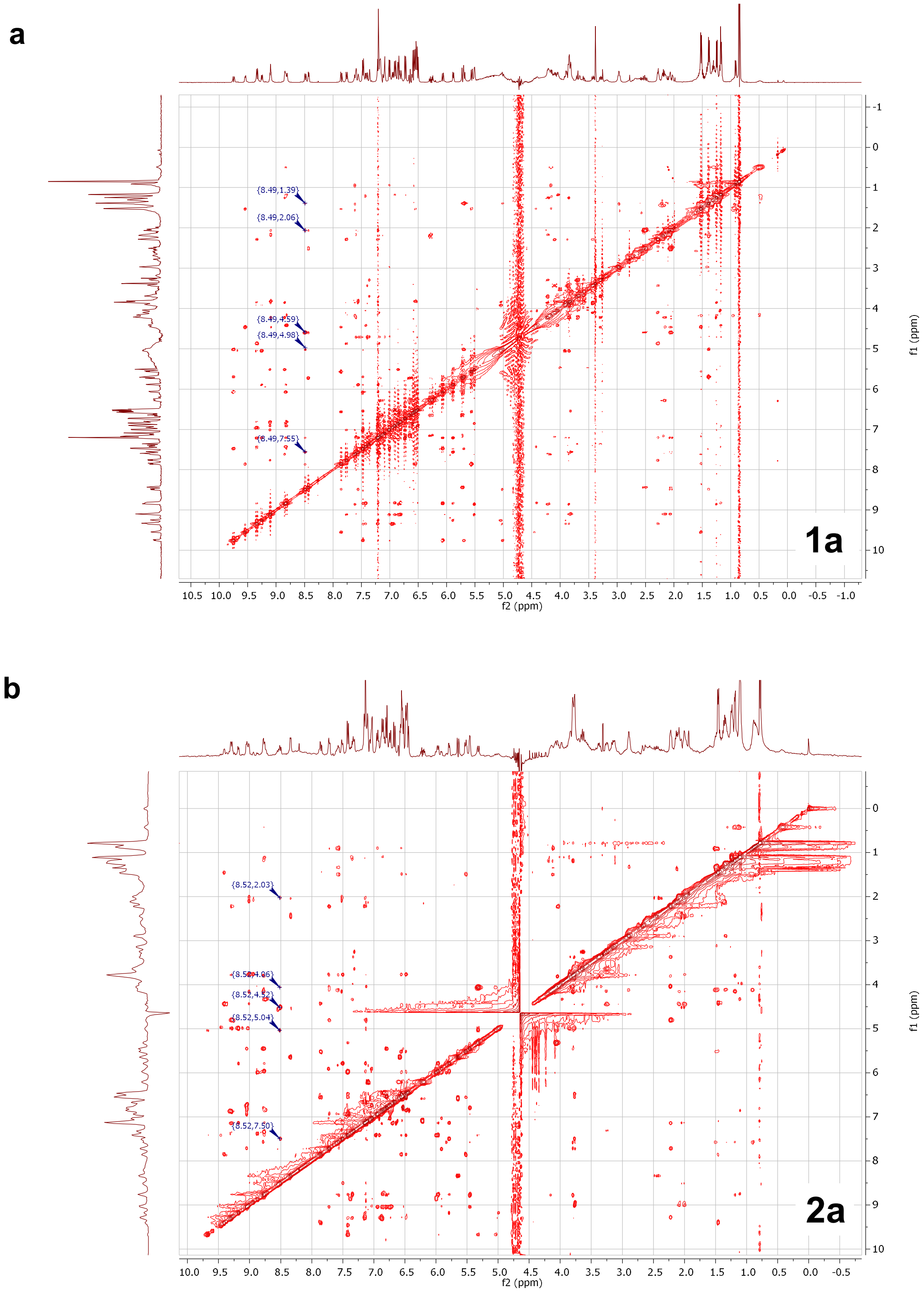

Supplementary Fig. 4. 2D NOESY spectra of 1a (a) and 2a (b) in H_2O-D_2O-TFA (9:1:0.05, v/v/v), acquired at 500 MHz.

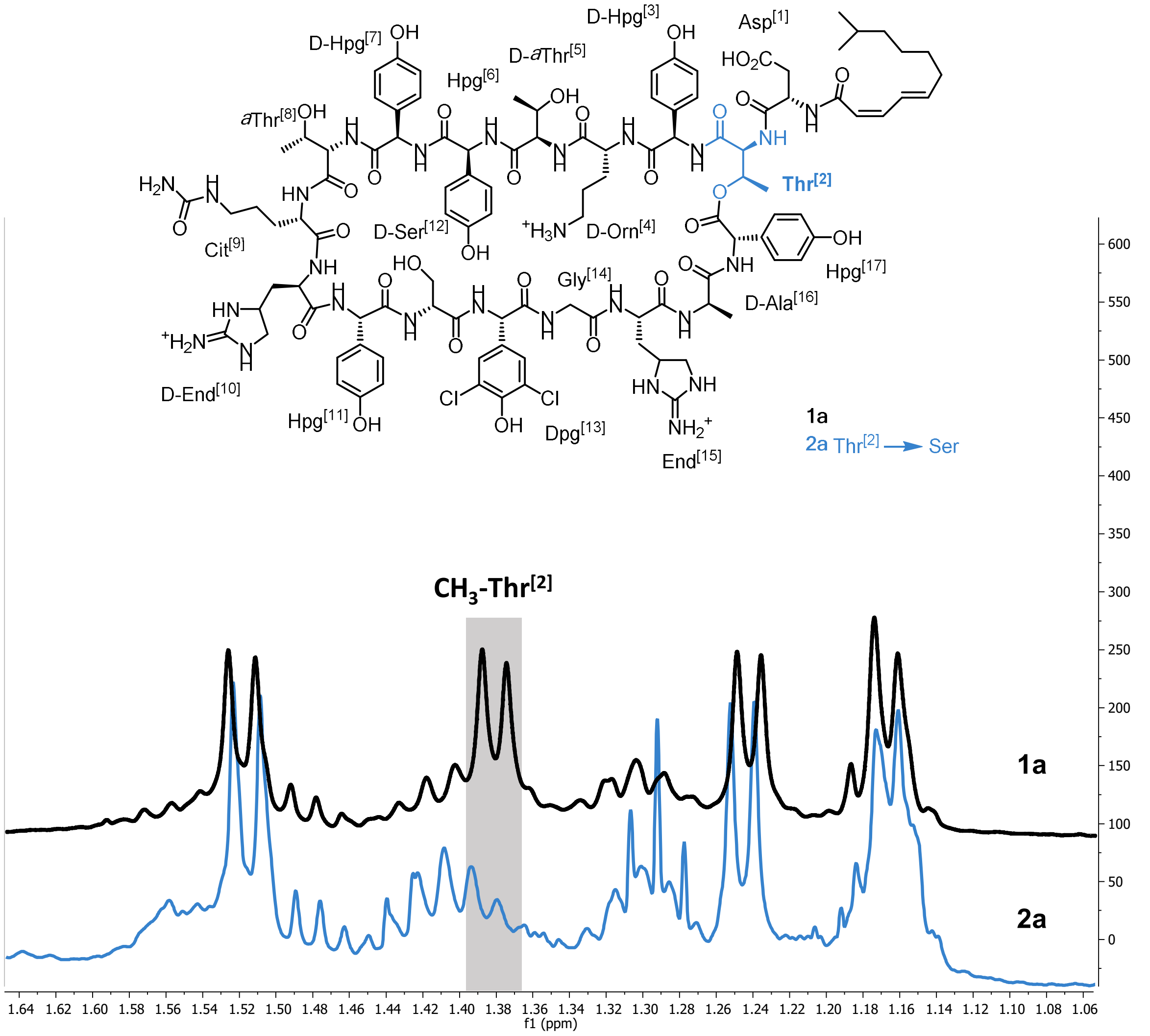

Supplementary Fig. 5. Enlarged section of the ^{1}H NMR spectra of 1a and 2a. Comparison of the proton spectra revealed the loss of a doublet at 1.39 ppm corresponding to the methyl moiety at Thr[2] in **1a**

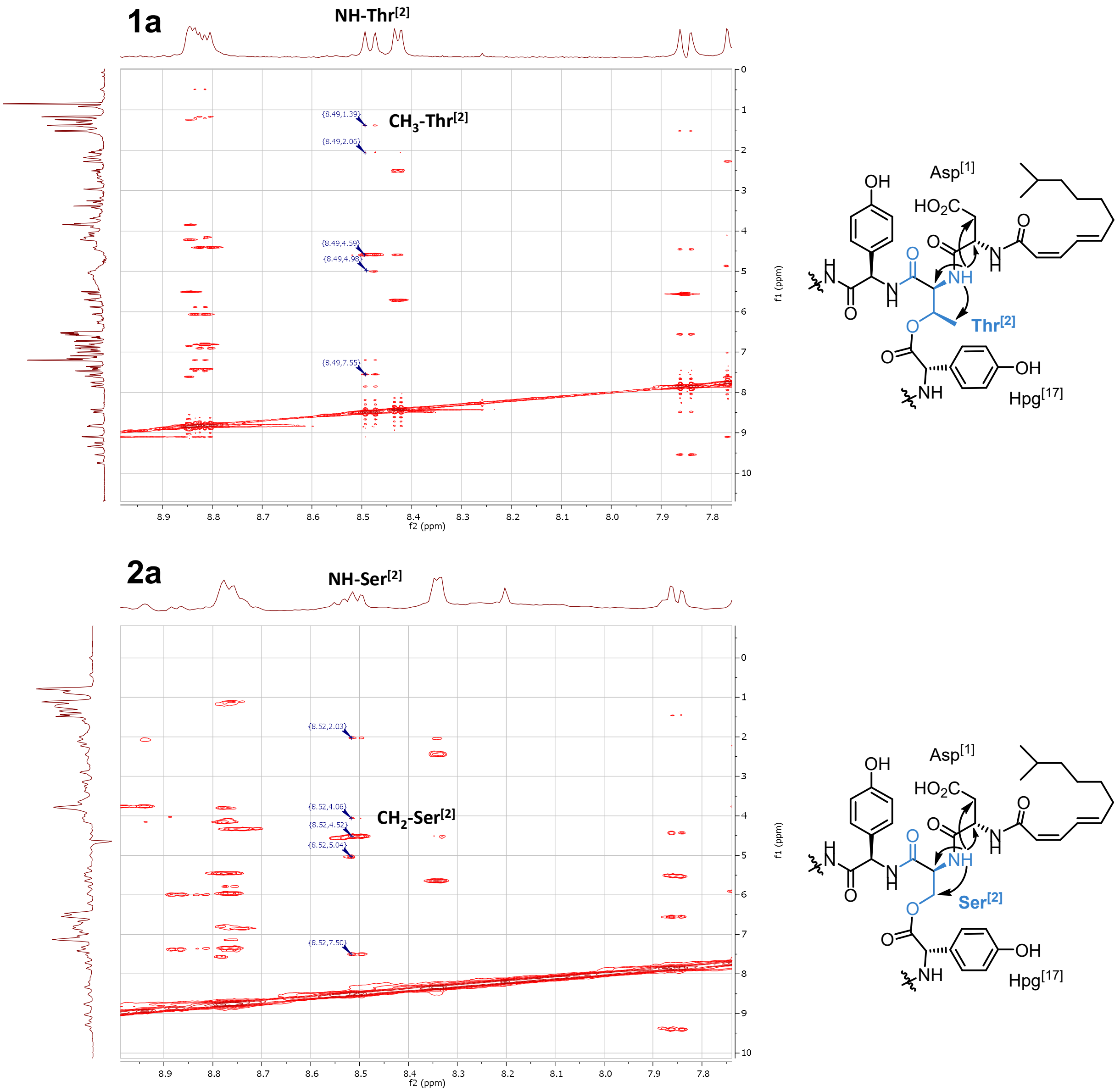

Supplementary Fig. 6. Enlarged section of the NOESY spectra of 1a and 2a, focusing on the NH moiety of residue 2. Comparison of correlations from the NH moiety revealed the loss of a methyl signal and the appearance of a new signal at 4.06 ppm corresponding to the methylene moiety in **2a.**

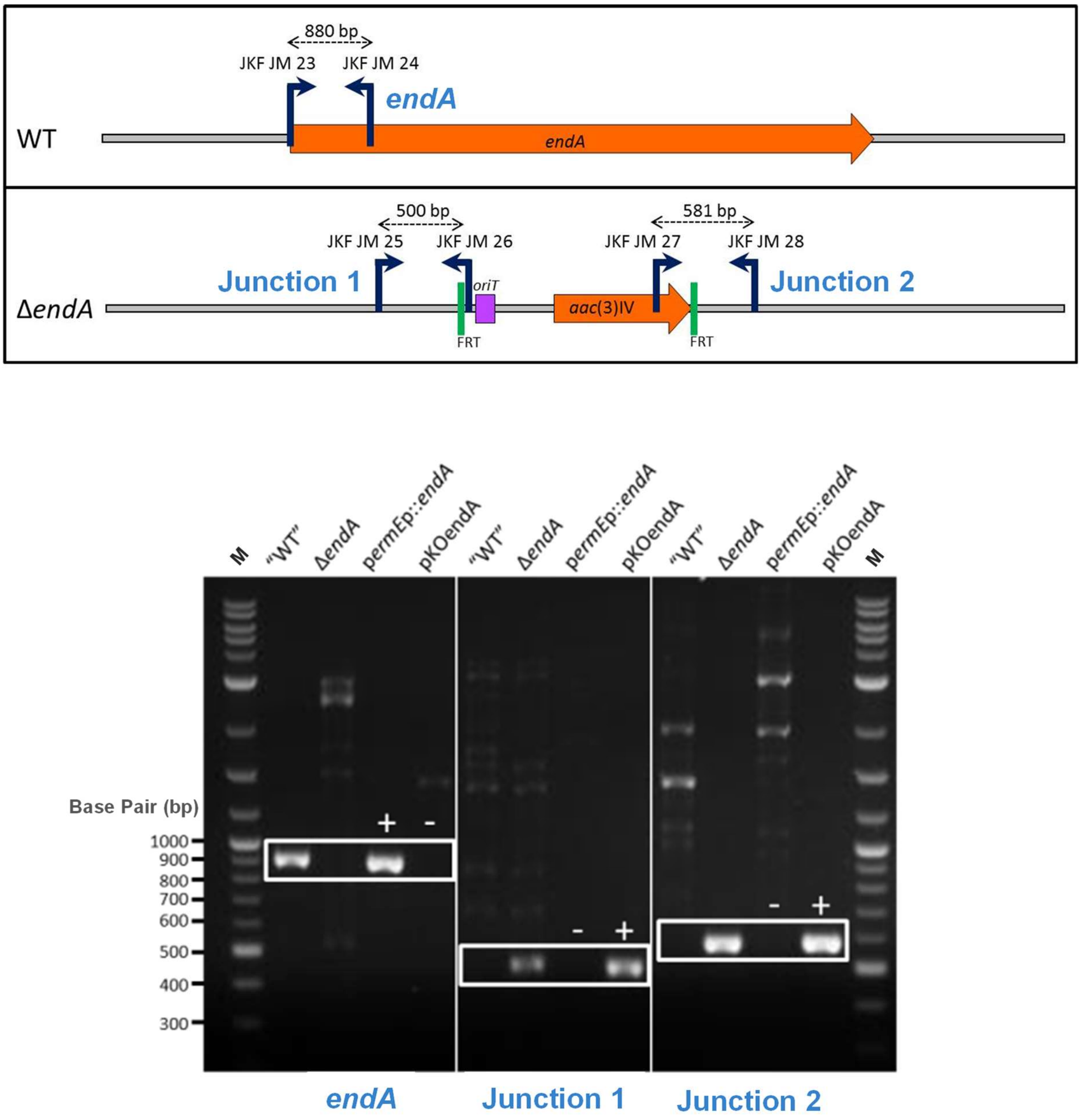

Supplementary Fig. 7. Homologous recombination mediated *endA* gene knock-out and gene complementation. Diagnostic PCR using the indicated primers to determine that *endA* has been replaced by an apramycin resistance cassette (*aac(3)IV*) in the *endA* deletion strain (Δ*endA*) and has been complemented in *endA* complementation strain (*permEp::endA*). Wild type (WT) and plasmid used for *endA* knock-out (pKOendA) were used as controls. This experiment was performed once.

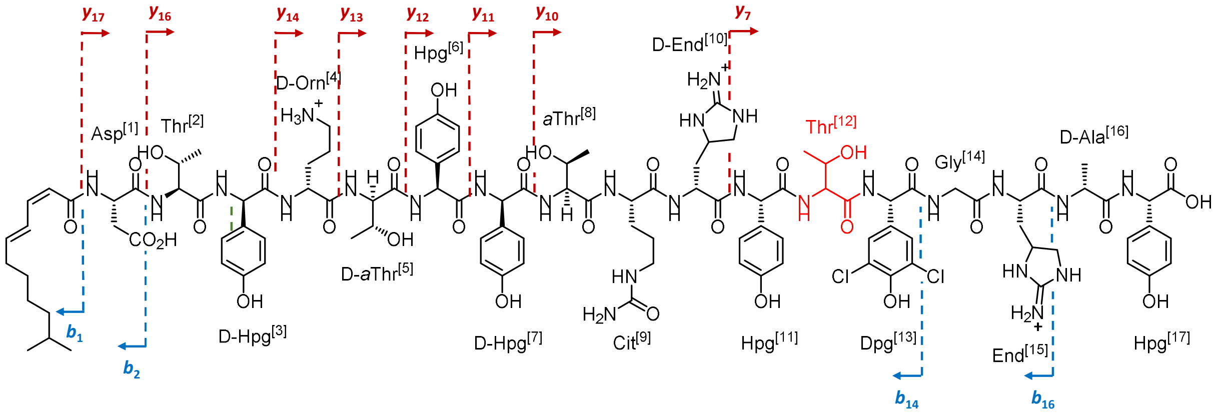

	3a (Ser[12] → Thr)					
	Calc' [M+H]⁺	Obs'd [M+H]⁺	Calc' [M+2H]²⁺	Obs'd [M+2H]²⁺	Calc' [M+3H]³⁺	Obs'd [M+3H]³⁺
**Parent**					790.327	790.326
y_{17}			1095.919	1095.919	731.280	731.277
y_{16}			1037.904	1137.907	692.272	692.273
y_{14}			921.861	921.862		
y_{13}			864.822	864.824		
y_{12}			814.298	814.302		
y_{11}			739.774	739.778		
y_{10}			666.249	666.257		
y_7	917.275	917.281				
$b_{14 (-FA, -CONH2, -H2O)}$			843.329	843.322		
$b_{16 (-FA)}$			978.887	978.861		
b_2	294.171	294.170				
b_1	179.143	179.143				

Supplementary Fig. 8. LC-HRMS/MS characterization of 3a produced by mutant F2 in this study.

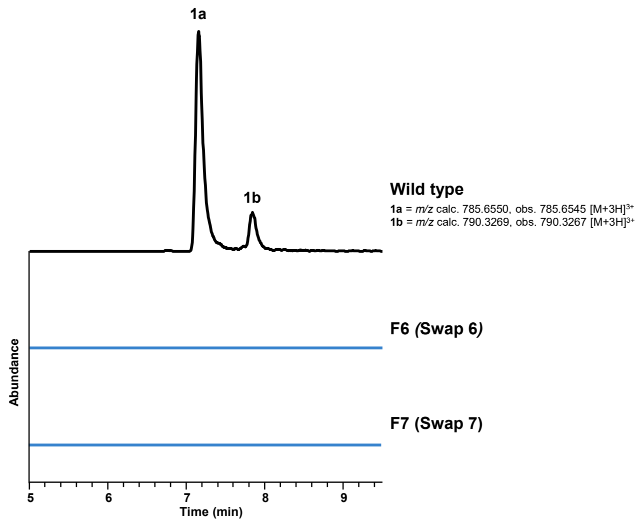

Supplementary Fig. 9. Extracted ion chromatographs from LC-HRMS analysis of extracts from *S. fungicidicus* strains used in this study. No production of enduracidin was observed in both F6 and F7 mutants.

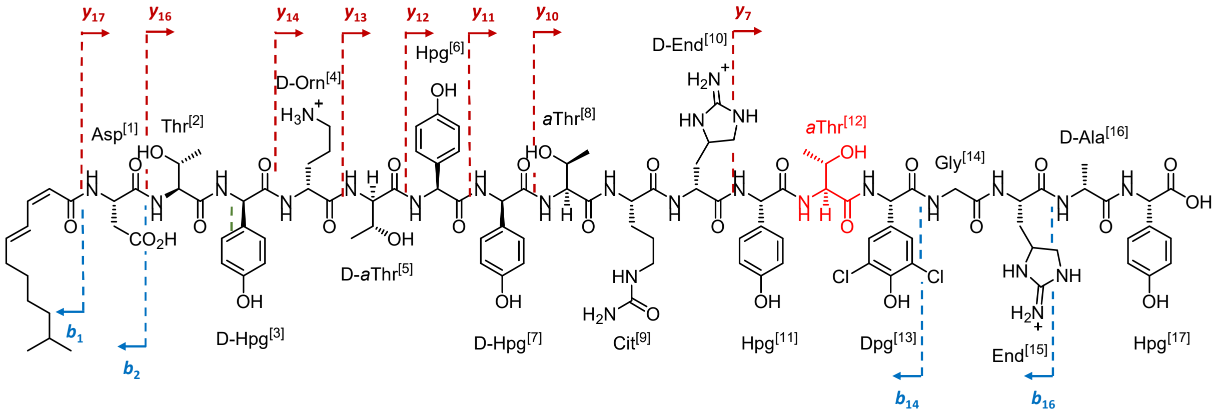

4a (Ser[12] → aThr)						
	Calc' $[M+H]^+$	Obs'd $[M+H]^+$	Calc' $[M+2H]^{2+}$	Obs'd $[M+2H]^{2+}$	Calc' $[M+3H]^{3+}$	Obs'd $[M+3H]^{3+}$
**Parent**					790.327	790.326
y_{17}			1095.919	1095.920	730.948	730.950
y_{16}			1037.904	1037.906	692.272	692.273
y_{14}			921.861	921.868		
y_{13}			864.822	864.822		
y_{12}			814.298	814.282		
y_{11}			739.774	739.789		
y_{10}			666.249	666.249		
y_7	917.275	917.277				
$b_{14\ (-FA,\ -CONH2,\ -H2O)}$			842.827	842.813		
$b_{16\ (-FA)}$			978.887	978.869		
b_2	294.171	294.171				
b_1	179.144	179.143				

Supplementary Fig. 10. LC-HRMS/MS characterization of 4a produced by mutant F3 in this study.

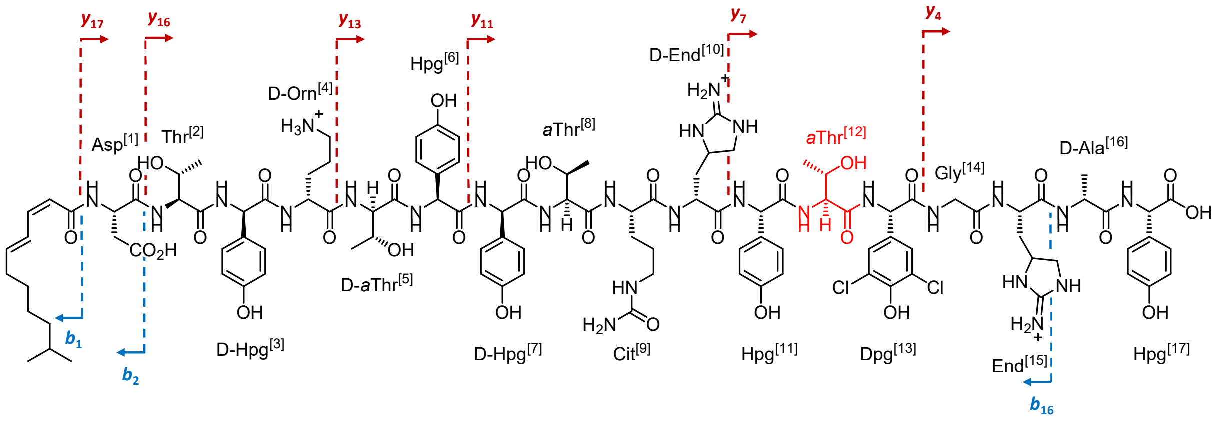

4a (Ser[12] → aThr)						
	Calc' [M+H]$^+$	Obs'd [M+H]$^+$	Calc' [M+2H]$^{2+}$	Obs'd [M+2H]$^{2+}$	Calc' [M+3H]$^{3+}$	Obs'd [M+3H]$^{3+}$
**Parent**					790.327	790.327
y_{17}			1095.919	1095.919		
y_{16}			1037.904	1037.906	692.272	692.271
y_{13}			864.822	864.822		
y_{11}			739.774	739.774		
y_7	917.275	917.270				
y_4	450.210	450.208				
$b_{16\ (-FA)}$			978.887	978.883		
b_2	294.171	294.1701				
b_1	179.144	179.143				

Supplementary Fig. 11. LC-HRMS/MS characterization of 4a produced by mutant F4 in this study.

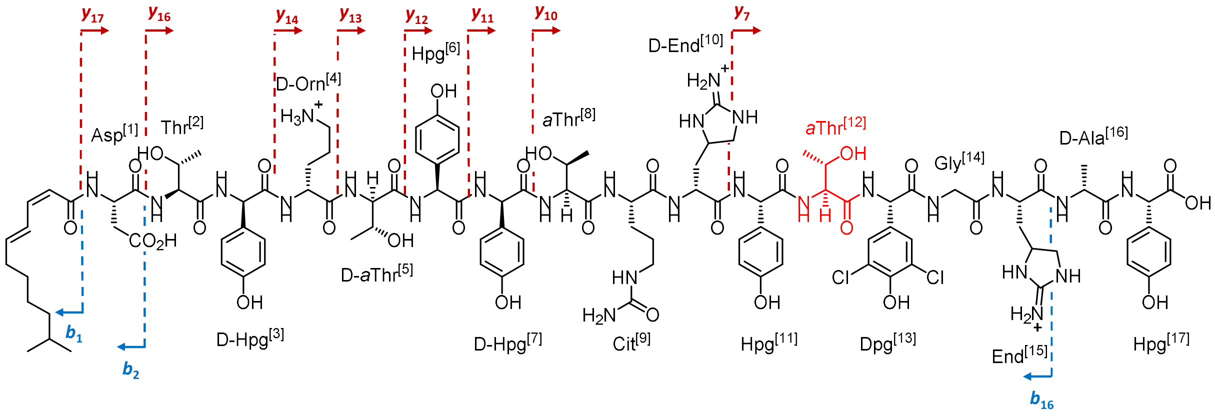

4a (Ser[12] → *a*Thr)						
	Calc' $[M+H]^+$	Obs'd $[M+H]^+$	Calc' $[M+2H]^{2+}$	Obs'd $[M+2H]^{2+}$	Calc' $[M+3H]^{3+}$	Obs'd $[M+3H]^{3+}$
**Parent**					790.327	790.327
y_{17}			1095.919	1095.919		
y_{16}			1037.904	1037.906	692.272	692.272
y_{14}			921.861	921.856		
y_{13}			864.822	864.823		
y_{12}			814.298	814.301		
y_{11}			739.774	739.776		
y_{10}			666.249	666.254		
y_7	917.275	917.271				
$b_{16\ (-FA)}$			978.887	978.870		
b_2	294.171	294.170				
b_1	179.144	179.143				

Supplementary Fig. 12. LC-HRMS/MS characterization of 4a produced by mutant F5 in this study.

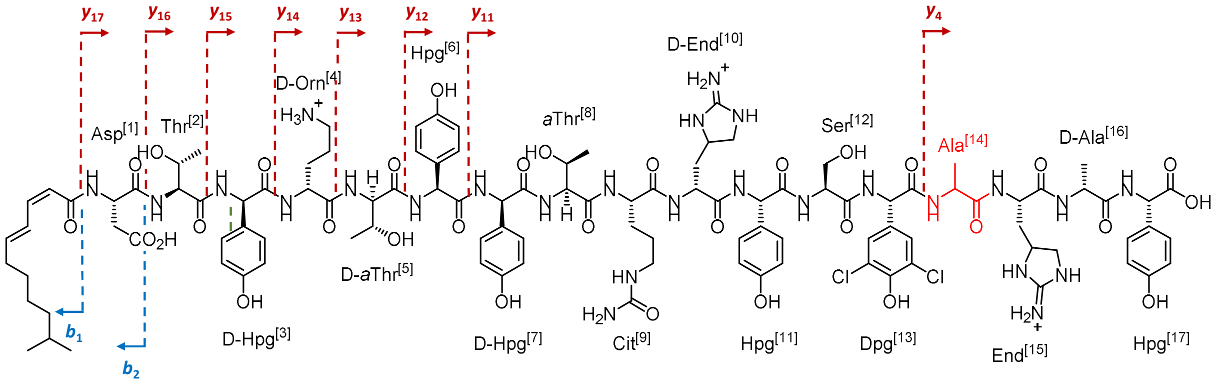

	Calc' [M+H]+	Obs'd [M+H]+	Calc' [M+2H]3+	Obs'd [M+2H]3+	Calc' [M+3H]3+	Obs'd [M+3H]3+
**5a (Gly[14] → Ala)**						
Parent					790.327	790.328
y_{17}			1095.919	1095.919		
y_{16}			1037.904	1037.906	692.272	692.273
y_{15}			996.385	996.380		
y_{14}			921.861	921.862		
y_{13}			864.822	864.823		
y_{12}			814.298	814.303		
y_{11}			739.774	739.771		
y_4	464.2252	464.2244				
b_2	294.171	294.171				
b_1	179.144	179.143				

Supplementary Fig. 13. LC-HRMS/MS characterization of 5a produced by mutant F8 in this study.

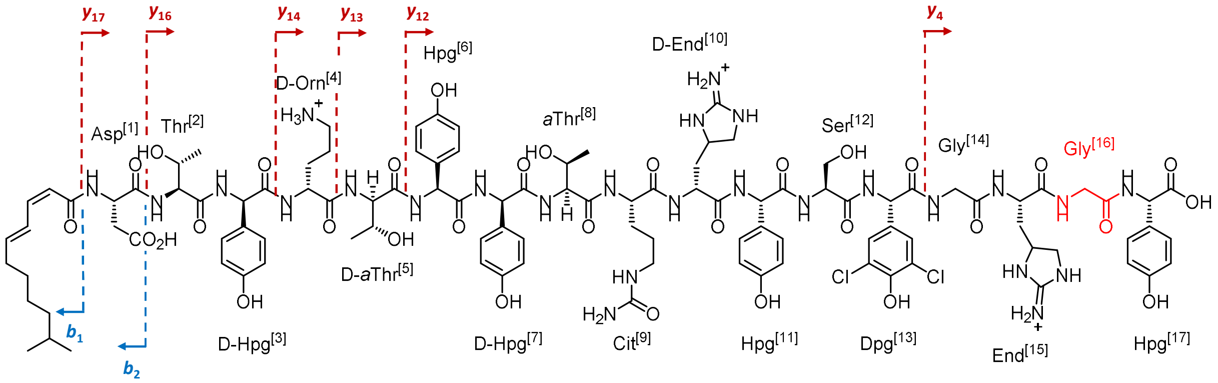

6a (Ala[16] → Gly)						
	Calc' [M+H]$^+$	Obs'd [M+H]$^+$	Calc' [M+2H]$^{3+}$	Obs'd [M+2H]$^{3+}$	Calc' [M+3H]$^{3+}$	Obs'd [M+3H]$^{3+}$
**Parent**					780.983	780.984
y_{17}			1081.903	1081.904	721.605	721.611
y_{16}			1023.888	1023.891	682.928	682.929
y_{14}			907.846	907.850		
y_{13}			850.806	850.806		
y_{12}			800.282	800.287		
y_4	436.194	436.196				
b_2	294.171	294.170				
b_1	179.144	179.143				

Supplementary Fig. 14. LC-HRMS/MS characterization of 6a produced by mutant F9 in this study.

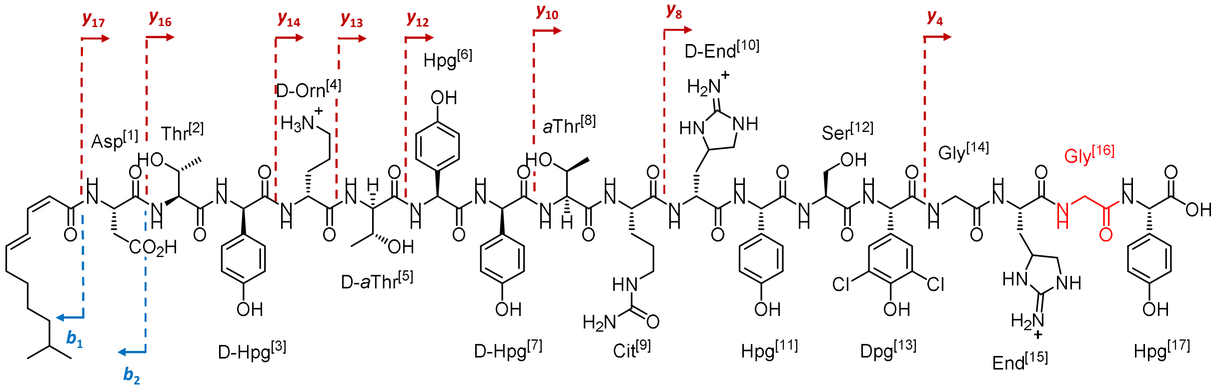

6a (Ala[16] → Gly)						
	Calc' $[M+H]^+$	Obs'd $[M+H]^+$	Calc' $[M+2H]^{3+}$	Obs'd $[M+2H]^{3+}$	Calc' $[M+3H]^{3+}$	Obs'd $[M+3H]^{3+}$
Parent					780.983	780.984
y_{17}			1081.903	1081.904	721.605	721.602
y_{16}			1023.888	1023.891	682.928	682.929
y_{14}			907.846	907.841		
y_{13}			850.806	850.808		
y_{12}			800.282	800.289		
y_{10}			651.234	651.242		
y_8			522.168	522.168		
y_4	436.194	436.195				
b_2	294.171	294.170				
b_1	179.144	179.143				

Supplementary Fig. 15. LC-HRMS/MS characterization of 6a produced by mutant F10 in this study.

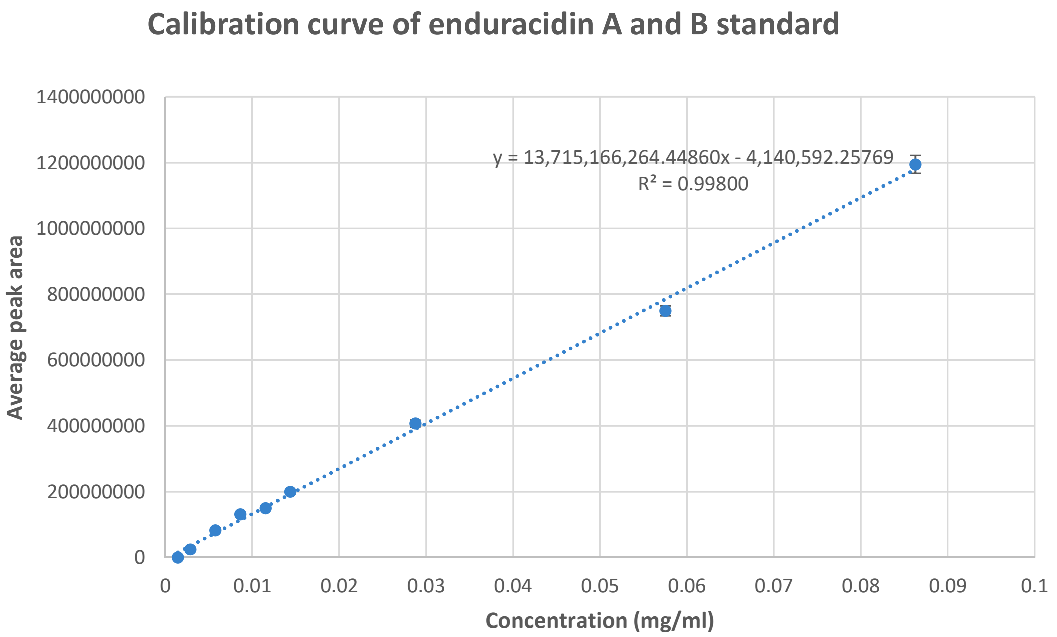

Supplementary Fig. 16. Calibration curve of commercial enduracidin a standard, plotted based on the peak area of the extracted ion chromatogram of m/z 785.6550 $[M+H]^+$. The experiment was performed in triplicate, n=3. Data are presented as mean values +/- standard error. Source data are provided with this paper.

Supplementary Table 1. Bacterial strains and plasmids used in this study.

**Strain/Plasmid**	**Characteristic(s)**	**Source/ Reference**
***Actinoplanes* sp. ATCC 33076**	Wild type strain, Ramoplanin-producing	ATCC
***Streptomyces coelicolor* M145**	Wild type strain, CDA-producing	ATCC
***Streptomyces* sp. DSM 40338**	Wild type strain, Pristinamycin-producing	DSMZ
***Pseudomonas syringae* DSM 10604**	Wild type strain, Syringafactin-producing	DSMZ
***Streptomyces griseolus* NRRL 3739**	Wild type strain, predicted to produce Streptobactin	NRRL
***Streptomyces rochei* NRRL B1559**	Wild type strain, predicted to produce Antimycin and Lipopeptide 8D1-1	NRRL
***Streptomyces rimosus sub. paramomycinus* NRRL 2455**	Wild type strain, predicted to produce Tyrobetaine	NRRL
Streptomyces fungicidicus		
**ATCC 21013**	Wild type strain, Enduracidin-producing	ATCC
**F1**	ATCC 21013 derivative with A domain swapping (Thr[2] → Ser[12])	This study
**Δ*endA***	Δ*endA* deletion mutant of ATCC 21013	This study
**Δ*endA*::*endA***	Δ*endA* mutant of ATCC 21013 complemented with *endA* (Δ*endA*::*endA*)	This study
**F1a**	Δ*endA* mutant of ATCC 21013 complemented with *endA* with A domain swapping (Thr[2] → Ser[12])	This study
**F2**	ATCC 21013 derivative with A domain swapping (Ser[12] →Thr[2])	This study
**F3**	ATCC 21013 derivative with A domain swapping (Ser[12] → *Allo*-Thr[5]_ Ramo13)	This study
**F4**	ATCC 21013 derivative with A domain swapping (Ser[12] → *Allo*-Thr[8]_ Ramo17)	This study
**F5**	ATCC 21013 derivative with A domain swapping (Ser[12] → *Allo*-Thr[12]_ Ramo14)	This study
**F6**	ATCC 21013 derivative with A domain swapping (Ser[12] → Ala[16])	This study
**F7**	ATCC 21013 derivative with A domain swapping (Ser[12] → Ala[16]_ Ramo14)	This study
**F8**	ATCC 21013 derivative with A domain swapping (Gly[14] → Ala[16])	This study
**F9**	ATCC 21013 derivative with A domain swapping (Ala[16] → Gly[14])	This study
**F10**	ATCC 21013 derivative with A domain swapping (Ala[16] → Gly[14]_ Ramo14)	This study
**F11**	ATCC 21013 derivative with A domain swapping (Thr[2] → Ser[1]_CDAPS1)	This study
**F12**	ATCC 21013 derivative with A domain swapping (Ser[12] →Thr[2]_Streptobactin)	This study
**F13**	ATCC 21013 derivative with A domain swapping (Gly[14] → Ala[4]_Tyrobetaine)	This study
**F14**	ATCC 21013 derivative with A domain swapping (Ala[16] → Gly[8]_Lipopeptide 8D1-1)	This study
**F15**	ATCC 21013 derivative with A domain swapping (Ser[12] →Thr[1]_Antimycin)	This study
**F16**	ATCC 21013 derivative with A domain swapping (Ser[12] →*Allo*_Thr[2]_Pristinamycin)	This study
**F17**	ATCC 21013 derivative with A domain swapping (Ser[12] →*Allo*_Thr[5]_Syringafactin)	This study
Escherichia coli		
**DH5α**	Host for general cloning	NEB
**ET12567 (pUZ8002)**	Donor strain for conjugation between *E.coli* and *Streptomyces*	*(1)*
**BW25113 (pIJ790)**	Strain for λ Red recombination system	*(2)*

**Plasmids**		
**pDWU5**	pBluescript KS+ based vector used for gene inactivation and gene complementation in this study	John Innes Centre
**pIJ86**	Template for ermE* promoter	John Innes Centre
**pIJ10700**	Template for hygromycin resistance cassette	John Innes Centre
**pSET152**	Integrative vector for gene complementation in this study	*(3)*
**pIJ773**	Template for apramycin resistance cassette	*(2)*
**pCRISPomyces-2**	*E.coli-Streptomyces* shuttle vector for gene inactivation in Streptomyces species	*(4)*
**pZY104**	pCRISPomyces-1 derivative for A domain swapping (Thr[2] → Ser[12])	This study
**pZY150**	pCRISPomyces-1 derivative for A domain swapping (Ser[12] → Thr[2])	This study
**pZY152**	pCRISPomyces-1 derivative for A domain swapping (Ser[12] → *a*Thr[5]_ Ramo13)	This study
**pZY153**	pCRISPomyces-1 derivative for A domain swapping (Ser[12] → *a*Thr[8]_ Ramo17)	This study
**pZY154**	pCRISPomyces-1 derivative for A domain swapping (Ser[12] → *a*Thr[12]_ Ramo14)	This study
**pZY187**	pCRISPomyces-1 derivative for A domain swapping (Ser[12] → Ala[16])	This study
**pZY188**	pCRISPomyces-1 derivative for A domain swapping (Ser[12] → Ala[16]_ Ramo14)	This study
**pZY167**	pCRISPomyces-1 derivative for A domain swapping (Gly[14] → Ala[16])	This study
**pZY163**	pCRISPomyces-1 derivative for A domain swapping (Ala[16] → Gly[14])	This study
**pZY164**	pCRISPomyces-1 derivative for A domain swapping (Ala[16] → Gly[14]_ Ramo14)	This study
**WL1**	pCRISPomyces-1 derivative for A domain swapping (Thr[2] → Ser[1]_CDAPS1)	This study
**WL15**	pCRISPomyces-1 derivative for A domain swapping (Ser[12] →Thr[2]_Streptobactin)	This study
**KR20**	pCRISPomyces-1 derivative for A domain swapping (Gly[14] → Ala[4]_Tyrobetaine)	This study
**AH22**	pCRISPomyces-1 derivative for A domain swapping (Ala[16] → Gly[8]_Lipopeptide 8D1-1)	This study
**KR19**	pCRISPomyces-1 derivative for A domain swapping (Ser[12] →Thr[1]_Antimycin)	This study
**AH11**	pCRISPomyces-1 derivative for A domain swapping (Ser[12] →*Allo*_Thr[2]_SnbC)	This study
**KR14**	pCRISPomyces-1 derivative for A domain swapping (Ser[12] →*Allo*_Thr[5]_Syringafactin)	This study

Supplementary Table 2. Bioinformatics information for each swap mutant

Mut.	Swap	A- domain	Sd size (aa)	Replacement A-domain	Sd size (aa)	Identity/Similarity (%)	
						A-domain	Sd
**F1**	1*	Thr[2]	139	Ser[12]	139	92/93	88/89
**F1a**	1†	Thr[2]	139	Ser[12]	139	92/93	88/89
**F2**	2*	Ser[12]	139	Thr[2]	139	92/93	88/89
**F3**	3*	Ser[12]	139	*Allo*-Thr[5] (Ramo13)	138	65/75	71/75
**F4**	4*	Ser[12]	139	*Allo*-Thr[8] (Ramo17)	143	54/63	55/65
**F5**	5*	Ser[12]	139	*Allo*-Thr[12] (Ramo14)	138	59/69	65/71
**F6**	6*	Ser[12]	139	Ala[16]	135	47/60	28/44
**F7**	7*	Ser[12]	139	Ala[16] (Ramo14)	133	48/61	31/48
**F8**	8*	Gly[14]	135	Ala[16]	135	75/82	75/83
**F9**	9*	Ala[16]	135	Gly[14]	135	75/82	75/83
**F10**	10*	Ala[16]	135	Gly[14] (Ramo14)	134	64/73	64/74
**F11**	11*	Thr[2]	139	Ser[1] (CDAPS1)	141	46/56	32/40
**F12**	12*	Ser[12]	139	Thr[2] (Streptobactin)‡	143	60/70	64/72
**F13**	13*	Gly[14]	135	Ala[2] (Tyrobetaine)‡	144	34/45	29/40
**F14**	14*	Ala[16]	135	Gly[8] (Lipopeptide 8D1-1)‡	135	53/65	44/59
**F15**	15*	Ser[12]	139	Thr[1] (Antimycin)‡	146	58/67	67/73
**F16**	16*	Ser[12]	139	Thr[2] (SnbC)	145	59/70	62/73
**F17**	17*	Ser[12]	139	*Allo*-Thr[5] (SyfB)	144	50/64	57/69

* Swap *via* CRISPR-cas9 editing
† Swap *via* gene complementation
‡ Predicted natural product and gene cluster
Shaded regions show successful swaps

Supplementary Table 3. Key proton chemical shifts (ppm) that identify each residues in enduracidin **a** and its new analogue.

	Reported-1a*[5]		This study-1a**		This study-2a**	
**Residue**	NH	CH_α	NH	CH_α	NH	CH_α
**Asp[1]**	8.14	4.58	8.43	4.5	8.41	4.57
**Thr[2]**	8.55	4.95	8.48	4.9	8.52 (Ser)	5.05 (Ser)
**Hpg[3]**	9.56	5.94	9.76	6.06	9.74	6.03
**Orn[4]**	8.73	4.05	8.82	3.85	8.83	3.86
**Thr[5]**	7.4	4.39	7.59	4.19	7.58	4.20
**Hpg[6]**	8.96	6.81	8.82	6.81	8.81	6.81
**Hpg[7]**	9.05	5.43	9.10	5.88	9.09	5.97
**Thr[8]**	-	3.77	7.55	3.8	7.56	3.84
**Cit[9]**	7.64	4.18	7.61	4.2	7.63	4.18
**End[10]**	7.73	-	7.76	4.8	7.78	4.87
**Hpg[11]**	9.38	6.94	9.33	6.94	9.33	6.93
**Ser[12]**	9.45	5.04	9.33	5.05	9.38	5.05
**Dpg[13]**	8.97	5.72	9.25	5.8	9.24	5.85
**Gly[14]**	7.73	3.87	8.82	4.2	8.83	4.15
**End[15]**	-	-	9.10	5.5	9.09	5.52
**Ala[16]**	9.68	4.38	9.54	4.44	9.46	4.48
**Hpg[17]**	7.94	5.44	7.85	5.5	7.92	5.59
**Side chain**	-	5.67	-	5.71	-	5.71

* H_2O-DMSO-*d6* (4:1, v/v)
** H_2O-D_2O-TFA (9:1:0.05, v/v/v)

Supplementary Table 4. Yield of enduracidin (**1a** & **1b**) and engineered variants in each cultivation flasks as estimated based on the standard curve of commercial enduracidin standard.

Production of enduracidin (1a & 1b) and engineered variants (a & b) in mg/L												
	**1**	**2**	**3**	**4**	**5**	**6**	**7**	**8**	**9**	**10**	**Average**	**Error**
**WT**	4.14	0.51	3.68	1.16	0.50	0.91	2.86	3.30	0.65	2.45	2.02	± 0.45
**F1**	0.93	2.77	0.49	0.63	2.90	1.78	0.68	2.64	0.45	1.12	1.44	± 0.32
**F2**	0.65	1.54	0.91	0.95	1.31	1.00	1.87	1.21	0.89	0.80	1.11	± 0.12
**F3**	0.35	0.36	0.62	0.42	0.43	0.49	0.41	0.50	0.66	0.85	0.51	± 0.05
**F4**	0.32	0.29	0.33	0.38	0.31	0.27	0.27	0.43	0.40	0.26	0.33	± 0.02
**F5**	0.48	0.66	0.75	0.61	0.39	0.58	0.55	0.53	0.84	1.01	0.64	± 0.06
**F8**	0.24	0.32	0.24	0.28	0.36	0.32	0.38	0.39	0.35	0.39	0.33	± 0.02
**F9**	0.49	0.32	0.43	0.28	0.51	0.38	0.37	0.33	0.59	0.26	0.40	± 0.03
**F10**	14.55*	1.91	1.41	0.89	15.68*	1.70	1.77	2.97	1.62	0.88	1.64	± 0.22
**WT****	1.32	2.66	2.37	-	-	-	-	-	-	-	2.11	± 0.41
**F12****	0.31	0.41	0.27	-	-	-	-	-	-	-	0.33	± 0.04
**F15****	0.46	0.71	1.83	-	-	-	-	-	-	-	1.00	± 0.42
**F16****	0.38	0.31	0.54	-	-	-	-	-	-	-	0.41	± 0.07
**F17****	0.87	0.77	0.55	-	-	-	-	-	-	-	0.73	± 0.09

* Statistical outlier

** Distinct batch of additional swap mutants

Supplementary Table 5. Mutations identified via whole genome sequencing

Strain	Position	SNV	Indel	Predicted gene affected	
				Accession number	Description
**F1**	3272110		C deleted	Intergenic region	
**F2**	*No mutations*				
**F7**	1696202-1696204		GAG inserted	WP_004933182.1	Insertion of Leu into iron chelate uptake ABC transporter family permease subunit CDS
	2785247	G to A		WP_121546049.1	Pro to leucine change in a helix-turn-helix domain-containing protein (transcriptional regulator)
	6732973		G inserted	WP_121547643.1	Change in reading frame of arginine deiminase CDS
**F8**	2862314	A to G		(1): WP_121546097.1<br>(2): N/A	2 overlapping genes: (1) silent mutation in a phosphatase PAP2 family protein CDS; (2) Lys to Arg in a hypothetical protein
	3299859	T to C		Intergenic region	
	3300293	A to G		WP_121546361.1	Phe to Ser, in a helix-turn-helix domain-containing protein(transcriptional regulator)
	3318273	G to A		WP_121548461.1	Leu to Phe in a hypothetical protein CDS
	3353049	A to G		WP_004930176.1	Asn to Asp in a M23 family metallopeptidase CDS
	3374078	A to G		WP_121546404.1	Glu to Gly in a NAD(P)/FAD-dependent oxidoreductase
	3380245	A to G		WP_004930124.1	Silent mutation in a sigma-70 family RNA polymerase sigma factor
	3391478	A to G		WP_163013272.1	Lys to Glu in a transporter CDS
	3432781	A to G		WP_121546444.1	Leu to Pro in a MFS transporter CDS
	3442305	G to A		WP_121546450.1	Arg to Cys in a molecular chaperone Hsp90
	3793404	G to A		WP_121546666.1	Ala to Val in a thiol reductant ABC exporter subunit CydD
	4550232		T insertion	Intergenic region	
	4550248		G insertion	Intergenic region	
	4984294		C deletion	WP_004926351.1	Change in reading frame of a PhoH family protein
**F9**	2442344		G inserted	Intergenic region	
	2543897	T to C		WP_121545921.1	Tyr to His in a helix-turn-helix domain-containing protein (transcriptional regulator)
	2747858	G to A		WP-163013249.1	Ala to Thr in a alpha/beta hydrolase CDS
	4097794		G deleted	N/A	Hypothetical protein coding sequence (will shift reading frame)
	4561287	C to T		WP_121547137.1	Glu to Lys in an RNA-directed DNA polymerase CDS
	4728880-4728888		Deletion of 9 bp (TGGACACCA)	WP_004926852.1	Deletion of 3 amino acids (Asp, Thr, Met) in a sucC gene
	5305688	T to C		WP_048459195.1	Start codon of a GntR family transcriptional regulator gene changed to a Thr
**F10**	2442339		G insertion	Intergenic region	
	5527161		C insertion	WP_121547644.1	Insertion (frame shift) in argF gene (ornithine carbamoyltransferase)

Supplementary Methods

Sequences of subdomains for Swaps 12-15

>FSD Thr[2] streptobactin*
```
ggggccgaggacgtctggacgctgttccactcctacgccttcgacttctcggtctgggagctgtggggtgccctgct
gcacggcggccggctggtcgtcgtcccgcacctgatcagccgcgacccggcggccttcctggagctgctcgcccgcg
agcgggtcaccgtgctcaaccagacgccgtccgccttctaccagctcagcgccgccgaccgggaccgccccggcagc
gaactcgccctgcgctacgtggtgttcggcggtgaggcgctcgaactcgcccggctggacgactggtacgaccggca
cgccgagcacgcccccacgctggtcaacatgtacggcatcaccgagaccaccgtgcacgtctcctacatcgccctgg
accgggcgagcgccgccgccgggagcagcagcaccatcggcgtc
```

>FSD Gly[14] tyrobetaine*
```
ggccccggctgccgcatgtcgcagaccttcggcctgaccttcgacccgtccgtgtacgacctgttcgtcgcgtgggg
ctccggcgccaccgtcgtcgtcccggacaaggacgagctgtaccggcccgtggactacgtggtacgccgccgcctca
cccactggttctccgtaccgtcgctggtcaccaccgccgagcggtacggcaatctgccgcctggccgcgtcaccacg
ctgcgccacagctgcttcagcgcggagccggtcaccgcgcagctgaccgcgcgctggcacgaggtcgcgcccggcag
ccgtatccacaacctgtacgggccgaccgaactgaccatcacctgcaccgaccacgaactcgccgacggcggagccg
ccgcggcgggcagcgccaacggcacggtgcctgtcggccggccgtac
```

>FSD Ala[16] lipopeptide 8D1-1*
```
accgacggcgaccggctgctcgccgtgaccacggtgggcttcgacatcgcgggtctggagatcttcctgccgctgct
gcacggggcggtgctggtgctcgcggacgaggagaccgcgaaggacccgcacgcgctgctgcgtgccgtgaccgcgt
cgggaatcaccatggtccaggcgacaccgagcctgtggcagggcgtcggggccgtggccgcggacgagctggccgga
gtacgcgtcctggtgggtggggaggcgctgccgcacgagctggcgcgcacgctgacggagcgggcccgttccgtgac
gaacctctacggtccgaccgaggtgacgatctgggccaccgccgccgacgtcgccgagtccgggccggtcatcggcg
tgccgctcgccaacacgtcg
```

>FSD Thr[1] antimycin*
```
cgggccgacgacgtgtggacgctgttccactcgtacgccttcgacttctccgtctgggagatctggggcgccctgct
gcacggcggccgggtcgtcgtcgtcccgtacgccgtcagccgctcgccccgggagttcctggagctggtgcaccgcg
aggggggtgaccgtcctcagccagaccccgtccgcgttcgagcagctcgtcgacgccgacctggagcggggcggggac
gccggggcgctgcgccacgtcgtcttcggcggcgaggccctgcgcccggcacggctgcgcccctgggccgaccgcca
cggcctggaccgcccggcgctggtgaacatgtacggcatcaccgagaccaccgtgcacgtcacccaccaccgcctca
cccgcgccgacctggacgacccgcgccgaggcagcgtcatcggcacccccctc
```

*Natural products are predicted using anti-SMASH

References

1. Gust, B., Kieser, T., & Chater, *PCR Targeting System in Streptomyces coelicolor A3(2)* (John Innes Centre, Norwich, 2002).

2. Gust, B., Challis, G. L., Fowler, K., Kieser, T., & Chater, K. F., PCR-targeted Streptomyces gene replacement identifies a protein domain needed for biosynthesis of the sesquiterpene soil odor geosmin. *Proc. Natl. Acad. Sci. U.S.A,* **100**, 1541-1546 (2003).

3. Kieser, T., Bibb, M. J., Buttner, M. J., Chater, K. F., & Hopwood, D. A. Practical Streptomyces Genetics. (John Innes Foundation, 2000).

4. Cobb, R. E., Wang, Y., & Zhao, H. High-efficiency multiplex genome editing of Streptomyces species using an engineered CRISPR/Cas system. *ACS Synth. Biol.* **4,** 723-728 (2015).

5. Castiglione, F., Marazzi, A., Meli, M. & Colombo, G. Structure elucidation and 3D solution conformation of the antibiotic enduracidin determined by NMR spectroscopy and molecular dynamics. *Magn. Reson. Chem.* **43**, 603-610, (2005).
