# Supplementary material for: Gene editing enables rapid engineering of complex antibiotic assembly lines
Source: Nat Commun. 2021 Nov 25;12:6872. doi: 10.1038/s41467-021-27139-1 (PMC8616955; doi:10.1038/s41467-021-27139-1)
Supplement: Supplementary file 2 — Description of Additional Supplementary Files [file 41467_2021_27139_MOESM2_ESM.pdf]

Title: Supplementary Data 1

Description: Primers used in cloning CRISPR-Cas9 constructs.
