# Supplementary material for: Gene editing enables rapid engineering of complex antibiotic assembly lines
Source: Nat Commun. 2021 Nov 25;12:6872. doi: 10.1038/s41467-021-27139-1 (PMC8616955; doi:10.1038/s41467-021-27139-1)
Supplement: Supplementary file 4 — Reporting Summary [file 41467_2021_27139_MOESM4_ESM.pdf]

nature research

Corresponding author(s): Jason Micklefield

Last updated by author(s): Oct 21, 2021

Reporting Summary

Nature Research wishes to improve the reproducibility of the work that we publish. This form provides structure for consistency and transparency in reporting. For further information on Nature Research policies, see our Editorial Policies and the Editorial Policy Checklist.

Statistics

For all statistical analyses, confirm that the following items are present in the figure legend, table legend, main text, or Methods section.

n/a	Confirmed	
☐	☒	The exact sample size (*n*) for each experimental group/condition, given as a discrete number and unit of measurement
☐	☒	A statement on whether measurements were taken from distinct samples or whether the same sample was measured repeatedly
☒	☐	The statistical test(s) used AND whether they are one- or two-sided<br>*Only common tests should be described solely by name; describe more complex techniques in the Methods section.*
☒	☐	A description of all covariates tested
☒	☐	A description of any assumptions or corrections, such as tests of normality and adjustment for multiple comparisons
☐	☒	A full description of the statistical parameters including central tendency (e.g. means) or other basic estimates (e.g. regression coefficient) AND variation (e.g. standard deviation) or associated estimates of uncertainty (e.g. confidence intervals)
☒	☐	For null hypothesis testing, the test statistic (e.g. *F*, *t*, *r*) with confidence intervals, effect sizes, degrees of freedom and *P* value noted<br>*Give P values as exact values whenever suitable.*
☒	☐	For Bayesian analysis, information on the choice of priors and Markov chain Monte Carlo settings
☒	☐	For hierarchical and complex designs, identification of the appropriate level for tests and full reporting of outcomes
☒	☐	Estimates of effect sizes (e.g. Cohen's *d*, Pearson's *r*), indicating how they were calculated

Our web collection on statistics for biologists contains articles on many of the points above.

Software and code

Policy information about availability of computer code

Data collection	Shimadzu LabSolutions, Thermo Xcalibur, Bruker Avance 500 MHz NMR
Data analysis	Thermo Xcalibur v2.2 (mass spectrometry), Shimadzu LabSolutions Lite v5 (HPLC), antiSMASH viewer v6.0 (gene cluster analysis), Clustal OMEGA web form and Vector NTI Suite 6(sequence alignments), MestReNova v11 (NMR), ChemDraw Professional v16 (chemical structures and exact mass calculations), HGAP4 algorithm SMRT link v8.0, Geneious Prime 2021.1

For manuscripts utilizing custom algorithms or software that are central to the research but not yet described in published literature, software must be made available to editors and reviewers. We strongly encourage code deposition in a community repository (e.g. GitHub). See the Nature Research guidelines for submitting code & software for further information.

Data

Policy information about availability of data

All manuscripts must include a data availability statement. This statement should provide the following information, where applicable:

- Accession codes, unique identifiers, or web links for publicly available datasets
- A list of figures that have associated raw data
- A description of any restrictions on data availability

Data availability: All data generated or analysed during this study are included in the Article, and its Supplementary Information, Supplementary Data file and Source Data file. Nucleotide sequences were obtained from GenBank and their accession numbers are as follows: enduracidin (DQ403252); ramoplanin (DD382878); CDA (CAB38518); pristinamycin (CAA67248); all other subdomain sequences are included in the Supplementary Methods. The original materials such as plasmids and strains that support the findings of this study are available from the corresponding author upon reasonable request.

Field-specific reporting

Please select the one below that is the best fit for your research. If you are not sure, read the appropriate sections before making your selection.

[x] Life sciences [ ] Behavioural & social sciences [ ] Ecological, evolutionary & environmental sciences

For a reference copy of the document with all sections, see nature.com/documents/nr-reporting-summary-flat.pdf

Life sciences study design

All studies must disclose on these points even when the disclosure is negative.

Sample size	No sample size calculation was used, sample size was chosen based on standard experiment method. Quantification of production was performed in at least triplicate and mean and standard deviation values calculated. For some production experiments more than 3 replicates were taken to ensure accuracy, and mean and standard deviation values calculated.
Data exclusions	The production for Swap 10 (Fig.6) was calculated from an average of 8 samples instead of 10, omitting two statistical outliers from Flask 1 and 5.
Replication	Experiments were carried out in at least triplicate and standard deviation provided. Each measurement is from a separate experiment but carried out at the same time. All attempts in replicates were successful.
Randomization	No randomization was applied due to the nature of this study where no animals or human subjects were involved. The sample size for analysis was small and all experiments provided quantitative results. Controls were always included in our experiments to avoid biased results
Blinding	No blinding was involved in this study as it does not involve animal or human subjects or group allocation.

Reporting for specific materials, systems and methods

We require information from authors about some types of materials, experimental systems and methods used in many studies. Here, indicate whether each material, system or method listed is relevant to your study. If you are not sure if a list item applies to your research, read the appropriate section before selecting a response.

Materials & experimental systems

n/a	Involved in the study	
[x]	[ ]	Antibodies
[x]	[ ]	Eukaryotic cell lines
[x]	[ ]	Palaeontology and archaeology
[x]	[ ]	Animals and other organisms
[x]	[ ]	Human research participants
[x]	[ ]	Clinical data
[x]	[ ]	Dual use research of concern

Methods

n/a	Involved in the study	
[x]	[ ]	ChIP-seq
[x]	[ ]	Flow cytometry
[x]	[ ]	MRI-based neuroimaging
